# Human genetic variation in *GLS2* is associated with development of complicated *Staphylococcus aureus* bacteremia

**DOI:** 10.1371/journal.pgen.1007667

**Published:** 2018-10-05

**Authors:** William K. Scott, Felix Mba Medie, Felicia Ruffin, Batu K. Sharma-Kuinkel, Derek D. Cyr, Shengru Guo, Derek M. Dykxhoorn, Robert L. Skov, Niels E. Bruun, Anders Dahl, Christian J. Lerche, Andreas Petersen, Anders Rhod Larsen, Trine Kiilerich Lauridsen, Helle Krogh Johansen, Henrik Ullum, Erik Sørensen, Christian Hassager, Henning Bundgaard, Henrik C. Schønheyder, Christian Torp-Pedersen, Louise Bruun Østergaard, Magnus Arpi, Flemming Rosenvinge, Lise T. Erikstrup, Mahtab Chehri, Peter Søgaard, Paal S. Andersen, Vance G. Fowler

**Affiliations:** 1 John P. Hussman Institute for Human Genomics, Miller School of Medicine, University of Miami, Miami, FL, United States of America; 2 Dr. John T. Macdonald Foundation Department of Human Genetics, Miller School of Medicine, University of Miami, Miami, FL, United States of America; 3 Department of Medicine, Division of Infectious Diseases, Duke University Medical Center, Durham, NC, United States of America; 4 Duke Clinical Research Institute, Duke University Medical Center, Durham, NC, United States of America; 5 Department of Bacteria, Parasites, and Fungi, Statens Serum Institut, Copenhagen, Denmark; 6 Department of Cardiology, Copenhagen University Hospital, Herlev-Gentofte, Denmark; 7 Department of Health Science and Technology, Aalborg University, Aalborg, Denmark; 8 Department of Clinical Microbiology, Copenhagen University Hospital, Rigshospitalet, Copenhagen, Denmark; 9 Department of Clinical Medicine, Copenhagen University Hospital, Rigshospitalet, Copenhagen, Denmark; 10 Department of Cardiology, Rigshospitalet and Department of Clinical Medicine, University of Copenhagen, Copenhagen, Denmark; 11 Department of Clinical Microbiology, Aalborg University Hospital, Aalborg, Denmark; 12 Department of Clinical Medicine, Aalborg University, Aalborg, Denmark; 13 Clinical Institute, Aalborg University, Aalborg, Denmark; 14 Department of Clinical Microbiology, Copenhagen University Hospital, Herlev-Gentofte, Denmark; 15 Department of Clinical Microbiology, Odense University Hospital, Odense, Denmark; 16 Department of Clinical Microbiology, Aarhus University Hospital, Aarhus, Denmark; 17 Department of Infectious Diseases, Copenhagen University Hospital, Hvidovre, Denmark; 18 Department of Cardiology, Aalborg University Hospital, Aalborg, Denmark; 19 Department of Veterinary and Animal Sciences, University of Copenhagen, Copenhagen, Denmark; Case Western Reserve University, UNITED STATES

## Abstract

The role of host genetic variation in the development of complicated *Staphylococcus aureus* bacteremia (SAB) is poorly understood. We used whole exome sequencing (WES) to examine the cumulative effect of coding variants in each gene on risk of complicated SAB in a discovery sample of 168 SAB cases (84 complicated and 84 uncomplicated, frequency matched by age, sex, and bacterial clonal complex [CC]), and then evaluated the most significantly associated genes in a replication sample of 240 SAB cases (122 complicated and 118 uncomplicated, frequency matched for age, sex, and CC) using targeted sequence capture. In the discovery sample, gene-based analysis using the SKAT-O program identified 334 genes associated with complicated SAB at p<3.5 x 10^−3^. These, along with eight biologically relevant candidate genes were examined in the replication sample. Gene-based analysis of the 342 genes in the replication sample using SKAT-O identified one gene, *GLS2*, significantly associated with complicated SAB (p = 1.2 x 10^−4^) after Bonferroni correction. In Firth-bias corrected logistic regression analysis of individual variants, the strongest association across all 10,931 variants in the replication sample was with rs2657878 in *GLS2* (p = 5 x 10^−4^). This variant is strongly correlated with a missense variant (rs2657879, p = 4.4 x 10^−3^) in which the minor allele (associated here with complicated SAB) has been previously associated with lower plasma concentration of glutamine. In a microarray-based gene-expression analysis, individuals with SAB exhibited significantly lower expression levels of *GLS2* than healthy controls. Similarly, Gls2 expression is lower in response to *S*. *aureus* exposure in mouse RAW 264.7 macrophage cells. Compared to wild-type cells, RAW 264.7 cells with *Gls2* silenced by CRISPR-Cas9 genome editing have decreased IL1-β transcription and increased nitric oxide production after *S*. *aureus* exposure. *GLS2* is an interesting candidate gene for complicated SAB due to its role in regulating glutamine metabolism, a key factor in leukocyte activation.

## Author summary

Complications from bloodstream infection with *Staphylococcus aureus (S*. *aureus)* are important causes of hospitalization, significant illness, and death. The causes of these complications are not well understood, but likely involve genetic factors rendering people more susceptible to such infections, differences in the bacteria that cause the infection, and the interactions between them. We examined the parts of the human genome that code for proteins to find variations that were more common in people with complicated *S*. *aureus* bacteremia (SAB), and identified one gene, called *GLS2*, in which variation is more common in complicated SAB cases than uncomplicated cases. Expression of *GLS2* is lower in people with SAB than controls and in mouse white blood cells exposed to *S*. *aureus*. *GLS2* encodes a protein that regulates the metabolism of glutamine, a regulatory process that activates white blood cells. These cells are very important in the immune response to *S*. *aureus* infection, and therefore genetic variants that might influence their growth are important potential genetic risk factors for complicated SAB.

## Introduction

*Staphylococcus aureus* is a significant human pathogen and leading cause of skin and soft tissue infection (SSTI) and bacteremia (SAB) in community and healthcare settings. Incidence of SAB ranges from 10 to 30 per 100,000 person-years in developed countries and may present as an “uncomplicated” bloodstream infection or as a “complicated” infection involving a device implant, infective endocarditis, or bone and joint infection [1]. The etiology of SAB is complex, involving host susceptibility, microbial virulence, and healthcare-associated factors [1].

Efforts to identify common host genetic factors underlying SAB initially examined biologically plausible candidate genes involved in the innate immune response in animal models and human samples (reviewed by [2]). Several genes have been implicated in mouse models of infection [3–5], but variation in these genes has not yet been associated with SAB in humans. In contrast, genome-wide screens of *S*. *aureus* infections in individuals of European ancestry [6] and SAB in individuals of African-American ancestry [7] have reproducibly associated *S*. *aureus* infections with common genetic variants in the class II region of the major histocompatibility complex (MHC).

In addition to influencing risk of developing SAB, host genetic factors may also contribute to development of “complicated” infections such as infective endocarditis (IE). These more severe infections have been associated with specific bacterial strains (clonal complexes (CC), defined by patterns on multi-locus sequence typing and *spa* typing). For example, the CC5 and CC30 clonal complexes are associated with increased risk of IE; however, host response to SAB due to these strains is variable, and not all individuals with CC5 or CC30-related SAB develop IE [8, 9]. Candidate gene studies have associated IE with variation in *IL6*, *IL1B* [10] and *TLR6* [11], although these findings considered multiple bacterial infections underlying IE and have yet to be replicated. Taken together, these observations suggest that host genetic susceptibility and microbial strain variation influence development of IE.

Hypothesizing that such host genetic susceptibility is due in part to variants in coding sequences of genes (e.g. variants leading to protein-coding changes that might disrupt gene function or host-microbe interaction), we conducted a two-stage study to identify variants associated with complicated SAB, selecting candidate genes in a whole-exome sequencing discovery stage followed by a custom-sequencing replication stage. Such an approach captures both common and rare coding sequence variants, including very rare variants not included on standard genotyping arrays. Genetic variants then can be analyzed for association with complicated SAB individually or in gene-based tests defined by function, location, and allele frequency. Subsequent gene expression studies in human whole blood samples and mouse cell lines examined changes in *GLS2* expression in the context of *S*. *aureus* infection. The results of this study implicate variants in the *GLS2* gene, which regulates plasma glutamine levels important for modulating the adaptive immune response as risk factors underlying development of complicated SAB.

## Results

### Whole exome sequencing of the discovery sample

The discovery sample of 168 individuals (84 complicated SAB, 84 uncomplicated, frequency matched by age (in deciles), sex, and bacterial clonal complex) is described in Table 1. The majority of the sample was male (65%), and average age was 59.1 years. All participants were white and non-Hispanic. By design, the majority of the sample was infected with strains of *S*. *aureus* previously associated with complicated SAB (CC5 or CC30, 72%). All individuals were white, non-Hispanic ethnicity, and little population stratification was detected by EIGENSTRAT analysis. None of the ten principal components extracted by EIGENSTRAT was significantly associated with complicated SAB in the discovery sample (p>0.05), and therefore these variables were not included in subsequent analyses to adjust for potential confounding by population stratification.

**Table 1:** Description of discovery (168 white individuals with SAB from Duke University Hospital) and replication (240 white individuals with SAB from Danish DANSAB study group) samples.

	Discovery sample(n = 168)		Replication sample(n = 240)	
	Complicated SAB (n = 84)	Uncomplicated SAB (n = 84)	Complicated SAB (n = 122)	Uncomplicated SAB (n = 118)
**Sex**				
Male	55 (65%)	55 (65%)	79 (65%)	79 (67%)
Female	29 (35%)	29 (35%)	43 (35%)	39 (33%)
**Age**				
16–29	6 (7%)	6 (7%)	4 (3%)	4 (3%)
30–39	5 (6%)	5 (6%)	5 (4%)	4 (3%)
40–49	7 (8%)	7 (8%)	12 (10%)	9 (8%)
50–59	21 (25%)	21 (25%)	18 (15%)	14 (12%)
60–69	19 (23%)	22 (26%)	29 (24%)	33 (28%)
70–79	18 (21%)	14 (17%)	28 (23%)	34 (29%)
80–93	8 (10%)	9 (11%)	26 (21%)	20 (17%)
**Mean age**	59.1 years	59.1 years	65.6 years	65.2 years
**Bacterial clonal complex**				
CC5	37 (44%)	43 (51%)	34 (28%)	31 (26%)
CC8	23 (27%)	23 (27%)	24 (20%)	23 (19%)
CC30	24 (29%)	18 (21%)	64 (52%)	64 (54%)

After sequence alignment, base calling, and quality control steps, 404,808 autosomal single nucleotide variants (SNV) were analyzed for association with complicated SAB, adjusting for age (in deciles), sex, bacterial clonal complex (CC5 and CC30 vs. CC8) and sequencing batch. No SNV was significantly associated with complicated SAB at a genome-wide corrected threshold (p<5 x 10^−8^) in the overall sample (S1 Fig) or when stratified by bacterial clonal complex (CC5 and CC30 separate from CC8 (S2 and S3 Figs)), and no inflation of SNV test statistics was observed on quantile-quantile plots and estimates of the genomic inflation factor (λ = 0.75 (overall; S4 Fig), 0.77 (CC5 and CC30; S5 Fig) and 0.83 (CC8; S6 Fig). Gene-based analysis using SKAT-O (allowing for cumulative independent effects of SNVs annotated as being in a gene by SeattleSeq) did not detect any significant associations after Bonferroni correction (p<2.5 x 10^−6^) for testing all variants in 20,000 genes or when restricting analysis to SNV annotated by SeattleSeq as missense, nonsense and splice-site variants. The top gene-based results (p<1 x 10^−4^) overall and in the subsets (CC5 and CC30, CC8) are presented in Table 2, and results for all genes are presented in S1 Table (overall), S2 Table (CC5 and CC30) and S3 Table (CC8). Slight inflation of test statistics was observed on quantile-quantile plots (overall; S7 Fig, CC5 and CC30; S8 Fig, and CC8; S9 Fig). Analysis restricted to functional variants weakened evidence of association at all top genes other than *LCMT2* in the CC5 and CC30 subset, and no genes were included among the top results solely by analysis of functional variants. Complete SKAT-O results using only functional variants and corresponding quantile-quantile plots are provided in S4 Table and S10 Fig (overall), S5 Table and S11 Fig (CC5 and CC30), and S6 Table and S12 Fig (CC8). Little inflation of test statistics was observed on quantile-quantile plots.

**Table 2:** Top results (p<1 x 10^−4^) from SKAT-O gene-based association analysis in the discovery sample, overall (adjusted for age, sex, and bacterial clonal complex) and stratified by bacterial clonal complex (adjusted for age and sex).

Gene	SNV	p-value
**Overall (n = 168), all variants**		
***CHRNA2***	17	4.6 x 10^−5^
***PLEC***	191	4.9 x 10^−5^
***GNPDA1***	16	6.5 x 10^−5^
***FAM153B***	8	7.4 x 10^−5^
***CDK3***	9	9.1 x 10^−5^
GLS2**	19	5.4 x 10^−3^
Overall (n = 168), functional variants*		
*No genes with p<10*^*−4*^		
**CC5 and CC30 subset (n = 122), all variants**		
***LCMT2***	10	2.5 x 10^−5^
***EFCAB4B***	53	6.1 x 10^−5^
GLS2**	14	0.18
CC5 and CC30 subset (n = 122), functional variants*		
***LCMT2***	5	3.1 x 10^−6^
**CC8 subset (n = 46), all variants**		
***FAM111B***	2	5.6 x 10^−6^
GLS2**	13	1.8 x 10^−3^
CC8 subset (n = 46), functional variants*		
*No genes with p<10*^*−4*^		

*functional variant = missense, nonsense (stop-gain or stop-loss), or splice-gain or loss

** *GLS2* is included for comparison with other top results in the discovery dataset.

The SKAT-O results overall and in the two subsets were used to identify the most significant genes for consideration in the replication phase. Starting with genes with p < 1 x 10^−4^ in at least one analysis, the list was expanded by including the next-most-significant genes from each subset until a 2 Mb capture set was generated. This occurred at p<3.5 x 10^−3^ and yielded a set of 334 genes. Eight additional biologically interesting candidate genes suggested by prior studies in humans and mice (*DUSP3*, *FGA*, *FGB*, *FGG*, *FN1*, *PSME3*, *SPINK5*, *TNFAIP8*) were added to this set for a final total of 342 genes that were captured and analyzed in the replication set (S7 Table). These 342 genes contained 8,915 variants detected in the discovery dataset.

### Custom capture and sequencing of the replication sample

The replication set of 240 individuals (122 with complicated SAB and 118 with uncomplicated SAB), frequency matched by the same covariates as the discovery sample, is described in Table 1. The sample was 66% male, and average age was 65.4 years. All participants were white and non-Hispanic, and 80% of the sample was infected by bacterial clonal complexes previously associated with IE (CC5 or CC30). The replication sample was thus comparable to the discovery sample in these respects, but slightly older and more likely to carry CC5 or CC30. Like the discovery sample, little population stratification was detected by EIGENSTRAT analysis. None of the ten principal components extracted by EIGENSTRAT was significantly associated with complicated SAB in the replication sample (p>0.05), and therefore these variables were not included in subsequent analyses to adjust for potential confounding by population stratification.

**Fig 1:**
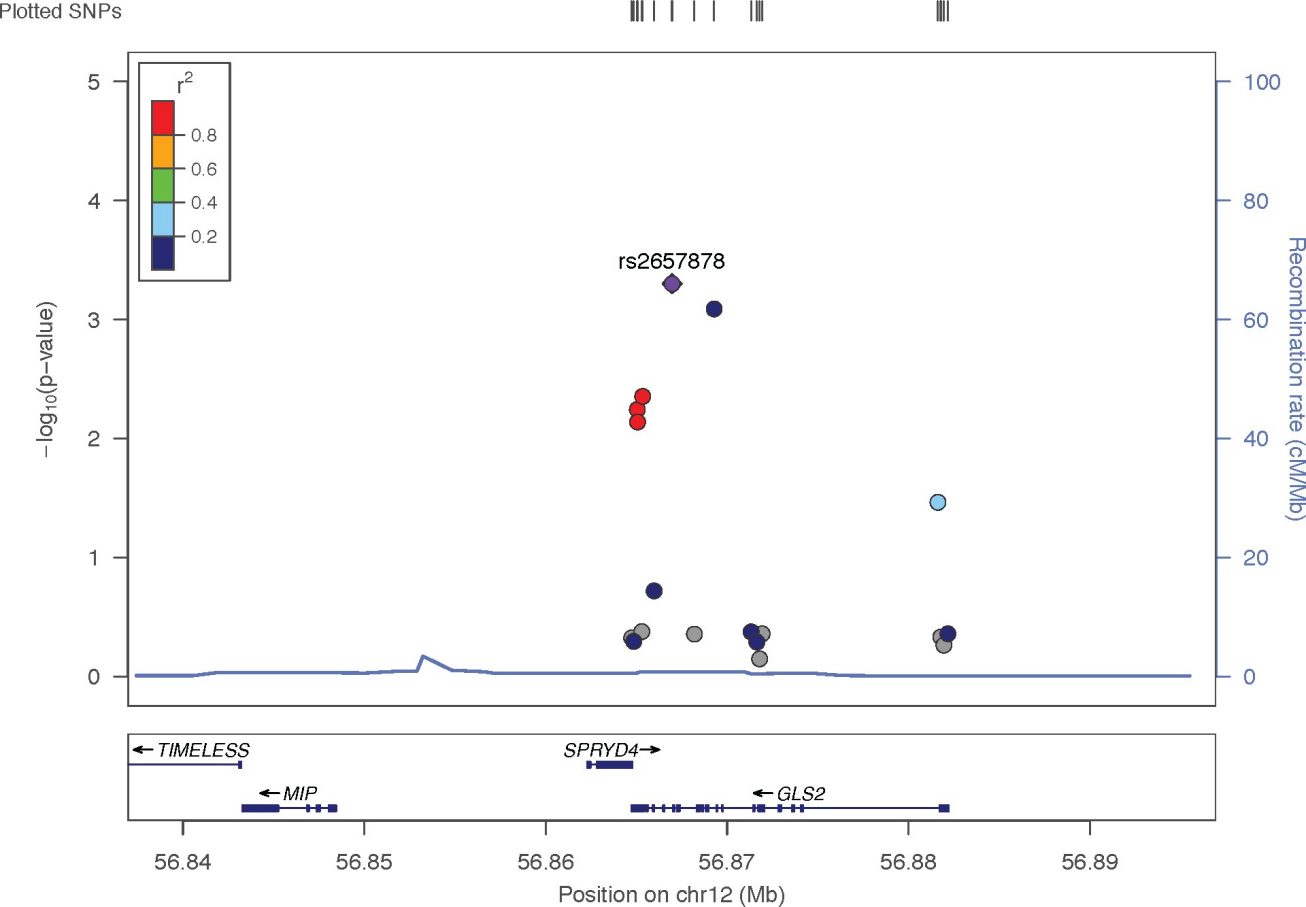
Regional association plot surrounding *GLS2* in the overall replication sample. The–log_10_ p-values for individual SNV association tests are plotted against chromosomal position. The strongest association is at intronic variant rs2657878 (purple diamond). The next strongest result is at rs937115 (blue circle), an intronic variant in modest linkage disequilibrium (r^2^<0.2) with rs2657878 in the 1000 Genomes November 2014 European sample. Three additional variants (red circles), including missense variant rs2657879, are in strong linkage disequilibrium (r^2^>0.8) with rs2657878.

After sequence alignment, base calling, and quality control steps, 10,931 single nucleotide variants (SNV) were analyzed for association with complicated SAB, adjusting for age (in deciles), sex, and bacterial clonal complex (CC5 and CC30 vs. CC8) (S13 Fig). No SNV was significantly associated with complicated SAB at a Bonferroni corrected threshold (p<4.5 x 10^−6^) in the overall sample or when stratified by bacterial clonal complex (CC5 and CC30, S14 Fig.; too few CC8 cases were included to analyze separately). No inflation of SNV test statistics was observed on quantile-quantile plots and estimates of the genomic inflation factor (λ = 0.70 (overall; S15 Fig), 0.71 (CC5 and CC30; S16 Fig). Gene-based analysis using SKAT-O in the overall replication sample detected significant association (p = 1.2 x 10^−4^) at one gene (*GLS2*) after Bonferroni correction for 342 genes tested (p<1.5 x 10^−4^). The top gene-based results (p<1 x 10^−2^) overall and in the CC5 and CC30 subset are presented in Table 3, and complete gene-based test results in the replication sample are presented in S8–S11 Tables, with corresponding quantile-quantile plots in S17–S20 Figs. Complete association results for individual SNV in the *GLS2* gene for both subsets and the meta-analysis are presented in S12 Table. While not significant after multiple testing correction, it is notable that the strongest overall association at an individual SNV in the replication sample is with intronic variant rs2657878 (p = 5 x 10^−4^) in *GLS2*, which is in strong linkage disequilibrium (r^2^ = 0.85) with missense variant rs2657879 (p = 4.4 x 10^−3^) (Fig 1, S21 Fig). While rs2657878 was not significantly associated with complicated SAB in the discovery dataset (p = 0.47; rank 262,339 of 404,809), the meta-analysis across subsets remained nominally significant (p = 2.4 x 10^−3^). The most significant *GLS2* result from the meta-analysis (which considered only SNV found in both subsets) was at less common (minor allele frequency 1.6%) intronic variant rs937115 (OR 8.01, p = 2.0 x 10^−4^), which was also not significant after multiple testing correction. When meta-analyzing gene-based test results across the discovery and replication datasets, *GLS2* remains the top-ranked gene (p = 7.9 x 10^−4^), with the sum test across 11 SNV more significant than the burden test (rho = 0). The gene-based and individual SNV tests were not significant when considering CC30 alone in the discovery and replication datsets, suggesting that the more significant overall results in the replication set are not attributable to the greater proportion of the sample carrying CC30.

**Table 3:** Top results (p<1 x 10^−2^) from SKAT-O gene-based association analysis in the replication sample, overall (adjusted for age, sex, and bacterial clonal complex) and restricted to bacterial clonal complex CC5 or CC30 (adjusted for age and sex).

Gene	SNV	p-value
***Overall (n = 240)*, *all variants***		
***GLS2***	20	1.2 x 10^−4^
***BEND5***	10	1.8 x 10^−3^
***GPRC6A***	15	2.7 x 10^−3^
***C14orf79***	29	4.6 x 10^−3^
***TUBD1***	12	5.1 x 10^−3^
*Overall (n = 240)*, functional variants*		
***CCDC108***	19	1.8 x 10^−3^
***LOC100129175***	10	3.0 x 10^−3^
***GLS2***	1	4.7 x 10^−3^
***AMOTL2***	9	4.8 x 10^−3^
***CC5 and CC30 subset (n = 193)*, *all variants***		
***GLS2***	18	2.3 x 10^−3^
***TUBD1***	10	2.7 x 10^−3^
***NAA15***	10	4.6 x 10^−3^
***SEPT8***	40	7.1 x 10^−3^
***YLPM1***	36	8.4 x 10^−3^
*CC5 and CC30 subset (n = 193)*, functional variants*		
***LOC100129175***	10	1.7 x 10^−4^
***CCDC108***	18	3.4 x 10^−4^
***AMOTL2***	8	5.8 x 10^−3^
***C14orf79***	3	6.3 x 10^−3^

*functional variant = missense, nonsense (stop-gain or stop-loss), or splice-gain or loss

### *GLS2* expression in SAB cases and mouse macrophages after *S*. *aureus* exposure

**Fig 2:**
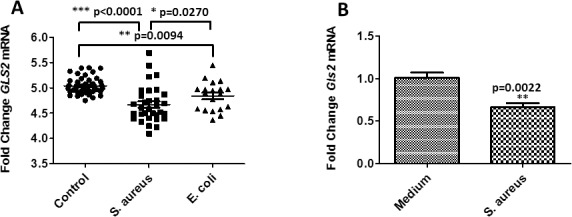
*GLS2* transcript is suppressed in (A) patients with *S*. *aureus* or *E*. *coli* blood stream infection and in (B) RAW 264.7 macrophages challenged with *S*. *aureus*. Data represent two independent experiments each with six biological replicates.

**Fig 3:**
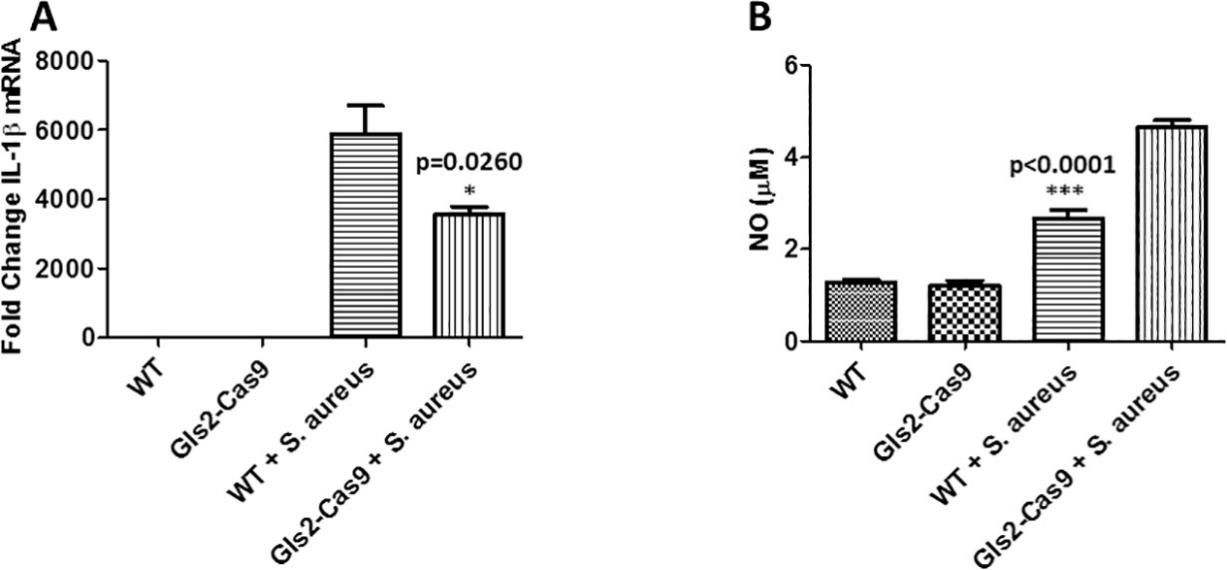
(A) Knockdown of *Gls2* decreased IL-1β mRNA and (B) enhanced NO production in RAW 264.7 macrophages challenged with *S*. *aureus*. Data represent two independent experiments, with six biological replicates for IL-1β (A) and three biological replicates for NO production (B).

To evaluate the clinical relevance of *GLS2* in human bloodstream infections, we compared microarray expression data from patients with *S*. *aureus* (n = 32) or *Escherichia coli* (n = 19) bloodstream infections (BSIs) against healthy controls (n = 44). We found that *GLS2* expression was significantly suppressed in *S*. *aureus* and *E*. *coli* BSI patients relative to healthy controls (Fig 2A), and that the significant difference in was present in both white and African-American subsets. Notably, no difference in expression in other genes adjacent to *GLS2* (*SPRYD4*, *MIP*, *RBMS2*) was observed, supporting a focus on that gene. To validate our microarray expression data, we next challenged RAW 264.7 macrophages with *S aureus* and measured *Gls2* expression by qRT-PCR. We observed the same pattern of *Gls2* suppression in macrophages challenged with *S aureus* (Fig 2B). Having shown that *GLS2* expression is suppressed in patients with *S*.*aureus* or *E*.*coli* BSI and in RAW 264.7 macrophages challenged with *S*. *aureus*, we next sought to understand the significance of the observed *GLS2* expression pattern. To this end, we used CRISPR-Cas9 technology to silence *Gls2* in RAW 264.7 macrophages, then challenged them with *S*. *aureus* and evaluated transcription of IL-1β, an important pro-inflammatory cytokine responsible for macrophage and neutrophil activation in response to *S*.*aureus* [12]. Silencing *Gls2* in RAW 264.7 macrophages significantly decreased IL-1β transcription compared to wild type (WT) cells (Fig 3A). Furthermore, the concentration of nitric oxide (NO), a prominent macrophage signaling molecule generated by inducible NO synthase, was significantly increased in *S*. *aureus Gls2*-silenced macrophage as compared to WT (Fig 3B). These data indicate that *GLS2* modulates innate immune responses to *S*. *aureus* stimulation both *in vitro* and *in vivo*.

## Discussion

This two-stage study utilized whole-exome sequencing to identify genes with differences in a discovery stage of patients with complicated and uncomplicated SAB, followed by a replication stage utilizing targeted capture and sequencing of 342 such genes. This strategy revealed a novel candidate gene for SAB, *GLS2*, in which multiple variants are more frequent in complicated SAB compared to uncomplicated SAB.

The initial gene-based test results in *GLS2* in the discovery dataset were nominally significant in multiple subsets, resulting in its inclusion in the replication study. However, no individual variant was significantly associated with complicated SAB in the discovery sample, indicating that the gene-based test result was due to the cumulative effect of multiple variants that did not have significant individual effects. The gene-based test of *GLS2* was the strongest result in the replication dataset, and several individual variants were nominally significantly associated with complicated SAB. The strongest single-variant association in the replication dataset was with a common variant (rs2657878) in intron 14 of *GLS2*. While this variant does not have a known functional consequence, it is in strong linkage disequilibrium with a coding-sequence variant in exon 18, rs2657879, which encodes a leucine to proline change at amino acid 581. The less common G allele at this variant has been reproducibly associated with lower plasma glutamine concentration in several genome-wide association studies of plasma metabolic markers [13–16] and was twice as frequent in complicated SAB cases in the replication sample (OR = 2.0, p = 0.004). These associations are biologically plausible, given the role of GLS2 (with GLS) in metabolizing glutamine. While some *GLS2* variants have been suggested to be eQTLs for the adjacent *SPRYD4* gene in GTEx data (www.gtexportal.org, accessed April 10, 2018), only *GLS2* was significantly differentially expressed in human BSI and was further shown to modulate response to *S*. *aureus* in mouse macrophages, suggesting that it is the most likely functional candidate gene. The observed pattern of association and inconsistent individual-variant results between discovery and replication samples suggest that the gene-based test might be an *indirect association*, whereby the coding-region variants are not themselves the biologically relevant variants, but are in linkage disequilibrium with as yet unidentified non-coding variants that might influence gene regulation and function. Therefore, the next steps in evaluating the role of GLS2 in SAB involved further examining gene expression and cellular response (cytokine production, nitric oxide production) in the context of exposure to S. aureus, and later examining regulatory sequence variants for association with these responses.

Existing gene expression studies in individuals with bacteremia showed that *GLS2* expression was lower in SAB cases compared to controls, and this pattern was reproduced in mouse RAW 264.7 cells. However, we were not able to examine the association between *GLS2* expression and complicated SAB, as sufficient RNA samples were not available in this dataset. Therefore, while these initial functional data demonstrate changes in *GLS2* expression in response to SAB, they do not fully explain the association of genetic variants with complicated SAB in particular. Studies designed to demonstrate differences in *GLS2* expression in complicated vs. uncomplicated SAB cases are needed, but sufficient numbers of RNA samples do not yet exist in the SABG and DANSAB datasets used for this study.

*GLS2* is an intriguing candidate gene for complicated SAB due to its role in glutamine metabolism, which is an important process for proliferation and activation of white blood cells such as neutrophils, macrophages and T-cells in response to *S*. *aureus* infection. Inhibition of GLS has been shown to reduce Th17 response [17], which is essential for effective neutrophil recruitment in response to *S*. *aureus* infection [18–20]. In mice, IL-17 is essential for host defense against *S*. *aureus* infections of the skin [21] and inhibition of IL-17 is associated with development of acute colitis after exposure to dextran sulfate sodium [22]. In humans, reduced levels of Th17 cells lead to increased susceptibility to *S*. *aureus* infections in patients with hyper-IgE syndrome [23, 24], atopic dermatitis [25], and mucocutaneous candidiasis [26]. Taken together, these findings suggest that poorer Th17 responses elicited by reduced GLS2 production may influence the development of complicated SAB through a less robust T-cell response to infection.

Further, nitric oxide, an endogenous signaling molecule produced by macrophages, is well known for its role in host defense mechanisms against various pathogenic bacteria [27]. When macrophages are challenged with tuberculosis for example, they produce NO which is converted into reactive nitrogen species (RNS) within infected macrophages resulting in bacterial death. The cytotoxic effects are thought to be indirect, and the role of NO is more complicated than a simple binary (on/off) response. In fact, when NO reacts with oxygen radicals, it generates peroxynitrite, nitrogen dioxide and dinitrogen trioxide that are highly toxic to the cells [28]. Consequently, NO levels are tightly regulated and the exact amount of NO produced determines whether an overall pro- or anti-inflammatory response predominates. In sepsis for example, increased production of NO triggers vasodilatation and consequent hypotension [29]. There is also prior evidence from *S*. *aureus* sepsis models implicating the level of NO generated in regulating neutrophil migration to the site of infection [30]. Here, we found that silencing *Gls2* not only results in overproduction of NO in macrophages challenged with *S*. *aureus* bioparticles but it also reduced expression of IL-1β relative to WT controls, suggesting that *Gls2* silencing alters macrophage responses to *S*. *aureus*.

Although *GLS2* has been suggested to act as a tumor suppressor gene, nothing is known about its role in *S*. *aureus* infection. Our results indicate that *Gls2* acts to regulate the amount of NO in *S*. *aureus*-challenged cells and thus protect those cells from damage induced by reactive NO byproducts. Consistent with our findings, it was reported that one of the functions of *Gls2* is to limit reactive oxygen species levels in cells and thus protect cells from oxidative stress-induced cell death [31, 32]. The fact that *GLS2* mutation is associated with complicated bacteremia could thus be the consequence of increased NO levels in these patients, resulting in the production of excessive cytotoxic oxygen radicals. Ochoa et al [33] similarly showed that high levels of circulating NO metabolites in the blood of general surgery patients with clinical sepsis correlated with severity of disease.

Another possible explanation could be reduced neutrophil migration to the site of infection which in turn hinders bacterial clearance [34]. Consistent with this, it was reported that high levels of NO inhibited neutrophil migration to the site of infection in a *S*. *aureus* sepsis model of infection [30, 35]. We also found that silencing of *Gls2* significantly reduced transcription of IL-1β, an important part of the innate immune response to *S*. *aureus*. IL-1β is required for both neutrophil recruitment [12] and regulation of pro-inflammatory Th17 response to *S*. *aureus* [36], and is sufficient for abscess formation in immunity against *S*. *aureus* in mice [37]. Taken together, these data strongly suggest a potential role of GLS2 in host susceptibility to *S*. *aureus* infection, whereby (as of yet not identified) variation in regulatory regions of the *GLS2* gene may alter gene expression in response to *S*. *aureus* infection, increasing NO and decreasing IL-1β, allowing complicated infection to develop.

While the association of *GLS2* variation with complicated SAB provides a novel target for additional study and potential intervention, there are caveats to the interpretation of these findings. The study was conducted in two samples of non-Hispanic whites of European descent, and therefore the results may not generalize to other populations. Also, while there is previous association of the L581P variant (rs2657879) with plasma glutamine concentration, a biological mechanism for this association has yet to be elucidated. Finally, this study did not detect association with other loci previously associated with endocarditis (*SLC7A14* [38], in a study that included a subset of the Danish sample used here in the non-significant replication), with risk of SAB (HLA class II region [6]), or with biologically plausible candidate genes identified from prior human and mouse studies (*DUSP3*, *FGA*, *FGB*, *FGG*, *FN1*, *PSME3*, *SPINK5*, *TNFAIP8)*. This is not surprising, as most of these genes (SLC7A14 being the exception) were associated with SAB overall, rather than complicated SAB in particular. Indeed, sub-analyses of prior studies did not find association between complicated SAB and HLA or other candidate genes. The lack of such associations might reflect lower power due to smaller sample sizes, or alternatively may indicate that genetic factors influencing initial development of SAB are distinct from those governing development of complicated infections such as endocarditis and/or bone and joint infection.

The association of *GLS2* variants with complicated SAB reinforces the conclusion that the strongest genetic susceptibility factors for *S*. *aureus* infection involved the adaptive immune response. Genome-wide association approaches in white [6] and African-American [7] samples reproducibly implicate the HLA class II region, which encodes cell surface molecules involved in antigen presentation and stimulation of the immune response to pathogens. Taken together, these results suggest that genetic susceptibility to SAB is influenced by several genetic variants that potentially modulate the macrophage and T-cell response to infection.

## Methods

### Ethics statement

The study was approved by the Duke University Institutional Review Board and participants recruited at Duke University provided written informed consent according to institutional policy. Patients dying from SAB prior to consent were included in the study in accordance with IRB-approved policies for decedent research. Danish samples were collected as a “treatment biobank” under protocols approved by the Danish Data Protection Agency (GEH-2014-053 // I-suite no 0337203372 and journal no. 2007-58-0015). Patient consent was not obtained for this study. As retrospective consent of treatment biobank participants for this specific study was not feasible, the Danish Regional Ethics Committee (journal no. H-4-2014-132) approved a waiver of consent for this study. As a condition of this approval, all samples were permanently anonymized prior to genetic analysis.

### Discovery dataset

The discovery dataset consisted of 168 individuals with monomicrobial SAB, selected from the *S*. *aureus* bacteremia group (SABG) repository [39], a prospective biobank of DNA, bloodstream microbial isolates, and clinical data from all individuals diagnosed with SAB and enrolled in the SABG repository since 1994 at Duke University Medical Center. As previously described in detail [9], individuals were classified as having complicated SAB if they had infective endocarditis ((IE) (native or device-associated)) or hematogenous bone and joint infection (vertebral osteomyelitis, septic arthritis) and were classified as uncomplicated SAB if no other types of complicated infection (meningitis, abscess, etc) were present. Exclusion criteria included outpatient status, age younger than 18 years, polymicrobial infection, and neutropenia. Equal numbers of complicated and uncomplicated SAB individuals (n = 84) were selected for study, frequency matched on age (in deciles), sex, and the clonal complex of the bloodstream *S*. *aureus* isolate. All individuals selected for study were white, non-Hispanic individuals of European descent.

### Replication dataset

A replication data set was created following the approach described for the discovery sample. Complicated cases (native or device-associated IE or hematogenous bone and joint infection) were matched to uncomplicated cases by age (in deciles), sex, and CC of the bloodstream isolate (CC 5, 8, or 30). In this way, a replication dataset of 240 patients was created from two sources. A total of 196 individuals were selected from the Danish Staphylococcal Bacteremia study group (DANSAB) biobank, a national resource of blood samples with over 2500 SAB cases maintained by the Statens Serum Institut and Herlev-Gentofte University Hospital which had been combined with clinical information from the Danish Staphylococcus aureus bacteremia registry [40]. An additional 44 SAB patients were identified by combining information from the national Danish Bacteremia Registry, patient journals and the Copenhagen Hospital Biobank [41]. Individuals were white, non-Hispanic (by definition) and of Northern European descent.

### Determination of bacterial clonal complexes

For the discovery sample, *spa* typing was used to infer clonal complex (CC) as previously described [8, 42]. Briefly, bacterial DNA was amplified using established PCR primers and sequences were determined via capillary electrophoresis. Sequences for *spa* were evaluated against eGenomics software (eGenomics, Inc, New York) and were used to determine CCs using a validated database [43]. For the replication sample, *spa* typing was used to classify isolates into clonal complexes, using similar laboratory methods and comparison to the MLST database (www.mlst.net) as previously described [40]. In both discovery and replication samples, only patients whose bloodstream isolate was unambiguously mapped to CC5, CC30, or CC8 were selected for this study.

### Whole exome sequencing (WES) of discovery dataset

Whole-exome capture was performed in four batches on genomic DNA isolated from peripheral blood leukocytes using the Agilent SureSelect 50Mb AllExon v5, including untranslated regions (UTRs), capture kit (Agilent, Santa Clara, CA). Samples were ‘barcoded’ for multiplex analysis and sequencing was performed with three samples pooled per lane on an Illumina HiSeq2000 instrument in the Center for Genome Technology, John P. Hussman Institute for Human Genomics, University of Miami. Sequence reads were assessed for quality and bases were called using the Illumina CASAVA 1.8 pipeline. Calls were then exported for alignment against the human reference genome (hg19) using the Burrows-Wheeler Alignment (BWA) software [44]. Variants were called using the GATK UnifiedGenotyper with VQSR recalibration [45]. Genotype calls with genotype quality <30, read depth <8, or Phred-scaled likelihood of reference genotype <99 were removed from analysis. Genotype variants with VQSR recalibration scores < = -2 were excluded. No allele frequency threshold was applied. After quality control, 404,808 variants were retained for analysis. SeattleSeq [46] version 138 was used to annotate variants to genes, and evaluate functional consequence (missense, nonsense, splice site variation).

### Statistical analysis of WES data

These variants were then analyzed for association with complicated SAB individually using Firth-bias corrected logistic regression [47], controlling for age, sex, clonal complex, and sequencing batch, as implemented in EPACTS (Efficient and Parallelizable Association Container Toolbox http://genome.sph.umich.edu/wiki/EPACTS). Cumulative effects of variants across a gene, controlling for the same covariates, were evaluated using SKAT-O specifying the optimal adjust option and small sample size adjustment [48]. With these parameters, SKAT-O either conducts a variant burden test, assuming all variants influence the trait in the same direction, or a weighted SKAT test, that gives variants with frequency less than 1% in the sample greater weight and allows for the presence of both rare risk and protective alleles. Analyses were conducted for the entire dataset (including all variants identified in the sequence captured by the whole exome capture kit, including some flanking intronic sequences and untranslated regions), as well as stratified by bacterial clonal complex (to allow for possible host-microbe interactions) and functional effect of variant (considering missense/nonsense/splice variants separately). A traditional genome-wide association significance level of p < 5 x 10^−8^ was used to evaluate statistical significance of individual variant tests and a Bonferroni-corrected threshold of p<2.5 x 10^−6^ (0.05/20,000 genes) was used for gene-based tests. Because no results were significant after multiple-testing correction, the top gene-based test results (with p-values < 10^−4^) are presented.

### Targeted capture and sequencing in the replication sample

Results of the gene-based SKAT-O analysis in the discovery sample were used to select targets for analysis in the replication sample. First, 334 genes with nominally significant gene-based results (p<3.5 x 10^−3^) overall or in one of the subset analyses (by clonal complex or functional status) were selected for analysis, and eight biological candidate genes were added based on results from mouse model studies. A targeted capture array for 342 genes (S7 Table) was designed using the Agilent SureSelect website (Agilent, Santa Clara, CA). Capture array probes were selected from the probes used in the whole exome capture (Agilent SureSelect AllExon 50 Mb + UTR v5), so that the sequences for these genes captured in the replication dataset were the same as those captured for the discovery dataset. Targeted capture was performed in a single batch on genomic DNA isolated from peripheral blood leukocytes. Samples were ‘barcoded’ for multiplex analysis and sequencing was performed with samples pooled 48 per lane on an lllumina HiSeq2500 instrument in the Center for Genome Technology, John P. Hussman Institute for Human Genomics, University of Miami. Sequence reads were assessed for quality and bases were called using the Illumina CASAVA 1.8 pipeline. Calls were then exported for alignment against the human reference genome (hg19) using the Burrows-Wheeler Alignment (BWA) software [44]. Variants were called using the GATK UnifiedGenotyper [45]. Genotype calls with genotype quality <30, read depth <8, or Phred-scaled likelihood of reference genotype <99 were removed from analysis. All variants, regardless of frequency, that passed these QC steps were retained for analysis. After quality control, 10,931 variants were retained for analysis. SeattleSeq [46] version 138 was used to annotate variants to genes, and evaluate functional consequence (missense, nonsense, splice site variation, scaled combined annotation dependent deletion (CADD) score [49].

### Statistical analysis of targeted capture data in the replication sample

As in the discovery sample, individual variants were analyzed for association with complicated SAB using Firth-bias corrected logistic regression [47], controlling for age, sex, and clonal complex, as implemented in EPACTS. Cumulative effects of variants across a gene were evaluated using SKAT-O specifying the optimal adjust option and small sample size adjustment [48]. Analyses were conducted for the entire dataset, as well as stratified by bacterial clonal complex (to allow for possible host-microbe interactions) and functional effect of variant (defined the same as for the discovery data set). Bonferroni multiple test corrections were applied to the single variant tests (p<4.5 x 10^−6^, 0.05/10,931 variants) and gene-based tests (p < 1.5 x 10^−4^, 0.05/342 genes). To evaluate consistency of results across the two samples, SKAT-O results were meta-analyzed across the two samples using the seqMeta package (https://github.com/DavisBrian/seqMeta). To summarize single-variant results across the most significant locus (*GLS2*) in both the replication and discovery datasets, we used LocusZoom[50] to create regional association plots of–log p-values for each variant in a 50kb window centered on *GLS2*, evaluating pairwise linkage disequilibrium and recombination rate using the 1000Genomes November 2014 EUR sample as a reference sample.

### Gene expression in human samples

Existing data on gene expression profiles in individuals with bloodstream infections were used to examine differences in *GLS2* and adjacent gene expression in SAB cases, *E*. *coli* bacteremia cases, and unaffected controls. Subjects were enrolled at Duke University Medical Center (DUMC; Durham, NC), Durham VAMC (Durham, NC), and Henry Ford Hospital (Detroit, Michigan) as part of a prospective, NIH-sponsored study to develop novel diagnostic tests for severe sepsis and community-acquired pneumonia. All participants were adults. All detail regarding clinical information of these patients, including age, gender as well as the microarray analysis has been previously published [51], and these data are publicly available (GSE33341). Gene expression results from that study for *GLS2*, *SPRYD4*, *MIP*, and *RBMS2* were examined for significant differences.

### Macrophage infection and RNA extraction

The RAW 264.7 mouse macrophage cell line was maintained at 37°C and 5% CO2 in Dulbecco’s Modified Eagle’s Medium (DMEM) supplemented with 10% Fetal Bovine Serum (FBS). A total of 5x10^5^ cells were pre-seeded in a 24-well plate for 24 h. *S*. *aureus* clinical strain, Sanger 476 was used for infection studies. *S*. *aureus* for infection was prepared exactly as described previously [5].

The cells were incubated with 5x10^6^ bacteria for 1 hour at 37°C. The non-phagocytized bacteria were removed by washing, and fresh medium was added. RNA was extracted at 5 hours post-infection using a Direct-zoL RNA MiniPrep kit (Zymo Research) according to the manufacturer’s instructions. The RNA was quantified using a Nanodrop 2000 instrument (Thermo Fisher Scientific). After quantification the RNA was reversed transcribed using High Capacity cDNA Reverse transcription kit (Thermo Fisher Scientific). Quantitative real-time PCR (qRT-PCR) was performed using SYBR Select Master Mix (Thermo Fisher Scientifc) and an ABI Prism 7500 Fast real-time PCR system (Life Technologies). The mRNA of *Gls2* was normalized to *Actin* rRNA. The *Gls2*, IL-1β and *Actin* primers used here are as follow: *Gls2* (5’-AAACGCCCCATCAGTTCAGT-3’/5’-AGGCTCTCCAAGGAAGTTGC-3’); *Actin* (5’-AGGTGTGATGGTGGGAATGG-3’/5’-GCCTCGTCACCCACATAGGA-3’). Il-1β (5’-GAGAACCAAGCAACGA-3’/5’-CAAACCGTTTTTCCATCTTCT-3’). Statistical analysis was performed with GraphPad Prism, version 5, software using a Mann-Whitney U nonparametric test.

### RAW Gls2 knockdown cell lines

To generate CRISPR/Cas9-mediated Gls2 knockdown RAW cells, we cloned sgRNAs targeting exon 2 or exon 6 of *Gls2* into LentiCRISPR.v2 (Addgene #52961), for coexpression of sgRNAs with *S*. *pyogenes* Cas9. Oligonucleotide primers sgRNA-1 (5’- ACCGTGGTGAACTTGTGGAT-3’), sgRNA-2 (5’- AGCGGCATGCTGCCTCGACT-3’) and sgRNA-3 (5’- GGCAGAAGGGGATCTTCGTG-3’) were ordered from Integrated DNA Technologies and cloned into LentiCRISPR as described (http://genome-engineering.org/gecko/). We prepared lentiviral particles for each sgRNA vector by cotransfecting HEK293T cells with the LentiCRISPR vector, psPAX2 and pMD2.g using TransIT-LT1 (Mirus) and harvesting virus-containing supernatant at 48 hours post transfection. RAW cells were transduced with virus at a multiplicity of infection (MOI) of <1 by spinfection in the presence of 8 ug/ml polybrene. Twenty-four hours post infection, cells were selected with 5 μg/ml puromycin for 72 hours and then expanded. Cells were harvested one-week post infection and genomic DNA was prepared (Qiagen QIAamp DNA Blood Mini kit). The *Gls2* locus was PCR amplified and assessed for editing using Surveyor assays (Integrated DNA Technologies) to confirm introduction of mutations in the gene.

### Measurement of nitric oxide in the supernatant of culture media

A total of 5x10^5^ cells pre-seeded in a 24-well plate for 24 h were treated with *S*. *aureus* bioparticles (Invitrogen) to a final concentration of 10 μg/ml. At 24 hours post-infection, supernatants were collected, and nitric oxide production was determined using Nitrate/Nitrite fluorimetric assay kit (Cayman) according to the manufacturer’s protocol.

## Supporting information

S1 Fig Manhattan plot of 404,808 single variant association results from Firth-bias corrected logistic regression in the overall discovery sample (n = 168). The–log_10_ p-values for each test are plotted against chromosomal position. A genome-wide significance threshold of 5 x 10^−8^ is indicated by the red horizontal bar.
(TIF)

S2 Fig Manhattan plot of 355,274 single variant association results from Firth-bias corrected logistic regression in the CC5 and CC30 subset of the discovery sample (n = 122). The–log_10_ p-values for each test are plotted against chromosomal position. A genome-wide significance threshold of 5 x 10^−8^ is indicated by the red horizontal bar.
(TIF)

S3 Fig Manhattan plot of 267,469 single variant association results from Firth-bias corrected logistic regression in the CC8 subset of the discovery sample (n = 46). The -log_10_ p-values for each test are plotted against chromosomal position. A genome-wide significance threshold of 5 x 10^−8^ is indicated by the red horizontal bar.
(TIF)

S4 Fig Quantile-quantile plot for single variant association results from the overall discovery sample. The black diagonal line indicates the expected distribution of test statistics under the null distribution. The red line indicates the linear trend of the ratio between observed and expected statistics. The plot indicates that overall test statistics are weaker than expected under the null hypothesis.
(TIF)

S5 Fig Quantile-quantile plot for single variant association results from the CC5 and CC30 subset of the discovery sample. The black diagonal line indicates the expected distribution of test statistics under the null distribution. The red line indicates the linear trend of the ratio between observed and expected statistics. The plot indicates that overall test statistics are weaker than expected under the null hypothesis.
(TIF)

S6 Fig Quantile-quantile plot for single variant association results from the CC8 subset of the discovery sample. The black diagonal line indicates the expected distribution of test statistics under the null distribution. The red line indicates the linear trend of the ratio between observed and expected statistics. The plot indicates that overall test statistics are weaker than expected under the null hypothesis.
(TIF)

S7 Fig Quantile-quantile plot for SKAT-O gene-based association results from the overall discovery sample. The black diagonal line indicates the expected distribution of test statistics under the null distribution. The red line indicates the linear trend of the ratio between observed and expected statistics. The plot shows little inflation of test statistics overall.
(TIF)

S8 Fig Quantile-quantile plot for SKAT-O gene-based association results from the CC5 and CC30 subset of the discovery sample. The black diagonal line indicates the expected distribution of test statistics under the null distribution. The red line indicates the linear trend of the ratio between observed and expected statistics. The plot shows little inflation of test statistics overall.
(TIF)

S9 Fig Quantile-quantile plot for SKAT-O gene-based association results from the CC8 subset of the discovery sample. The black diagonal line indicates the expected distribution of test statistics under the null distribution. The red line indicates the linear trend of the ratio between observed and expected statistics. The plot shows slight inflation of test statistics at nominal significance levels, but less inflation of test statistics at higher significance levels.
(TIF)

S10 Fig Quantile-quantile plot for SKAT-O gene-based association results using only functional (missense, nonsense, and splice) variants from the overall discovery sample. The black diagonal line indicates the expected distribution of test statistics under the null distribution. The red line indicates the linear trend of the ratio between observed and expected statistics. The plot shows little inflation of test statistics overall.
(TIF)

S11 Fig Quantile-quantile plot for SKAT-O gene-based association results using only functional (missense, nonsense, and splice) variants from the CC5 and CC30 subset of the discovery sample. The black diagonal line indicates the expected distribution of test statistics under the null distribution. The red line indicates the linear trend of the ratio between observed and expected statistics. The plot shows little inflation of test statistics overall.
(TIF)

S12 Fig Quantile-quantile plot for SKAT-O gene-based association results using only functional (missense, nonsense, and splice) variants from the CC8 subset of the discovery sample. The black diagonal line indicates the expected distribution of test statistics under the null distribution. The red line indicates the linear trend of the ratio between observed and expected statistics. The plot shows little inflation of test statistics overall.
(TIF)

S13 Fig Manhattan plot of 10,931 single variant association results from Firth-bias corrected logistic regression in the overall replication sample (n = 240). The–log_10_ p-values for each test are plotted against chromosomal position. A Bonferroni-corrected significance threshold of 4.5 x 10^−6^ is indicated by the blue horizontal bar; the traditional genome-wide significance threshold of 5 x 10^−8^ is indicated by the red horizontal bar.
(TIF)

S14 Fig Manhattan plot of 9,940 single variant association results from Firth-bias corrected logistic regression in the CC5 and CC30 subset of the replication sample (n = 193). The–log_10_ p-values for each test are plotted against chromosomal position. A Bonferroni-corrected significance threshold of 4.5 x 10^−6^ is indicated by the blue horizontal bar; the traditional genome-wide significance threshold of 5 x 10^−8^ is indicated by the red horizontal bar.
(TIF)

S15 Fig Quantile-quantile plot for single variant association results from the overall replication sample. The black diagonal line indicates the expected distribution of test statistics under the null distribution. The red line indicates the linear trend of the ratio between observed and expected statistics. The plot indicates that overall test statistics are weaker than expected under the null hypothesis.
(TIF)

S16 Fig Quantile-quantile plot for single variant association results from the CC5 and CC30 subset of the replication sample. The black diagonal line indicates the expected distribution of test statistics under the null distribution. The red line indicates the linear trend of the ratio between observed and expected statistics. The plot indicates that overall test statistics are weaker than expected under the null hypothesis.
(TIF)

S17 Fig Quantile-quantile plot for SKAT-O gene-based association results from the overall replication sample. The black diagonal line indicates the expected distribution of test statistics under the null distribution. The red line indicates the linear trend of the ratio between observed and expected statistics. The plot shows a slight but acceptable inflation of nominally significant test statistics.
(TIF)

S18 Fig Quantile-quantile plot for SKAT-O gene-based association results from the analysis of functional variants only in the replication sample. The black diagonal line indicates the expected distribution of test statistics under the null distribution. The red line indicates the linear trend of the ratio between observed and expected statistics. The plot shows little inflation of test statistics.
(TIF)

S19 Fig Quantile-quantile plot for SKAT-O gene-based association results from the CC5 and CC30 subset of the replication sample. The black diagonal line indicates the expected distribution of test statistics under the null distribution. The red line indicates the linear trend of the ratio between observed and expected statistics. The plot shows little inflation of test statistics.
(TIF)

S20 Fig Quantile-quantile plot for SKAT-O gene-based association results from analysis of functional variants only in the CC5 and CC30 subset of the replication sample. The black diagonal line indicates the expected distribution of test statistics under the null distribution. The red line indicates the linear trend of the ratio between observed and expected statistics. The plot shows little inflation of test statistics.
(TIF)

S21 Fig Regional association plot surrounding *GLS2* in the overall discovery sample. The–log_10_ p-values for individual SNV association tests are plotted against chromosomal position. Linkage disequilibrium is estimated from the 1000 Genomes 2014 European (EUR) sample. The strongest replication result at intronic variant rs2657878 is indicated by the purple diamond. No individual SNV tests are significant in *GLS2* or surrounding genes.
(TIF)

S1 Table SKAT-O results for 19,822 genes analyzed in the overall discovery sample. Results are presented in rank-order from most significant to least significant.
(CSV)

S2 Table SKAT-O results for 19,727 genes analyzed in the CC5 and CC30 subset of the discovery sample. Results are presented in rank-order from most significant to least significant.
(CSV)

S3 Table SKAT-O results for 19,466 genes analyzed in the CC8 subset of the discovery sample. Results are presented in rank-order from most significant to least significant.
(CSV)

S4 Table SKAT-O results for 15,619 genes analyzed using functional variants only in the overall discovery sample. Results are presented in rank-order from most significant to least significant.
(CSV)

S5 Table SKAT-O results for 14,984 genes analyzed using functional variants only in the CC5 and CC30 subset of the discovery sample. Results are presented in rank-order from most significant to least significant.
(CSV)

S6 Table SKAT-O results for 13,053 genes analyzed using functional variants only in the CC8 subset of the discovery sample. Results are presented in rank-order from most significant to least significant.
(CSV)

S7 Table Targeted capture array probe details for 342 target genes with SKAT-O association p<0.0035 in discovery sample analysis. Target regions spanned 2.05 Mb. Probes were selected from the Agilent SureSelect AllExon 50Mb + UTR v6 catalog, using the same parameters used to select that catalog and extending 10 bases from the ends of each. Targeted intervals are mapped to hg19 (GRCh37).
(DOCX)

S8 Table SKAT-O results for 342 genes analyzed in the overall replication sample. Results are presented in rank-order from most significant to least significant.
(CSV)

S9 Table SKAT-O results for 342 genes analyzed in the CC5 and CC30 subset of the replication sample. Results are presented in rank-order from most significant to least significant.
(CSV)

S10 Table SKAT-O results for 299 genes analyzed using functional variants only in the overall replication sample. Results are presented in rank-order from most significant to least significant.
(CSV)

S11 Table SKAT-O results for 289 genes analyzed using functional variants only in the CC5 and CC30 subset of the replication sample. Results are presented in rank-order from most significant to least significant.
(CSV)

S12 Table *GLS2* single variant association results from Firth bias-corrected logistic regression in the discovery and replication samples and meta-analysis results across samples. (XLSX)
