# Supplementary figures and images for: Human genetic variation in GLS2 is associated with development of complicated Staphylococcus aureus bacteremia
Source: PLoS Genet. 2018 Oct 5;14(10):e1007667. doi: 10.1371/journal.pgen.1007667 (PMC6192642; doi:10.1371/journal.pgen.1007667)

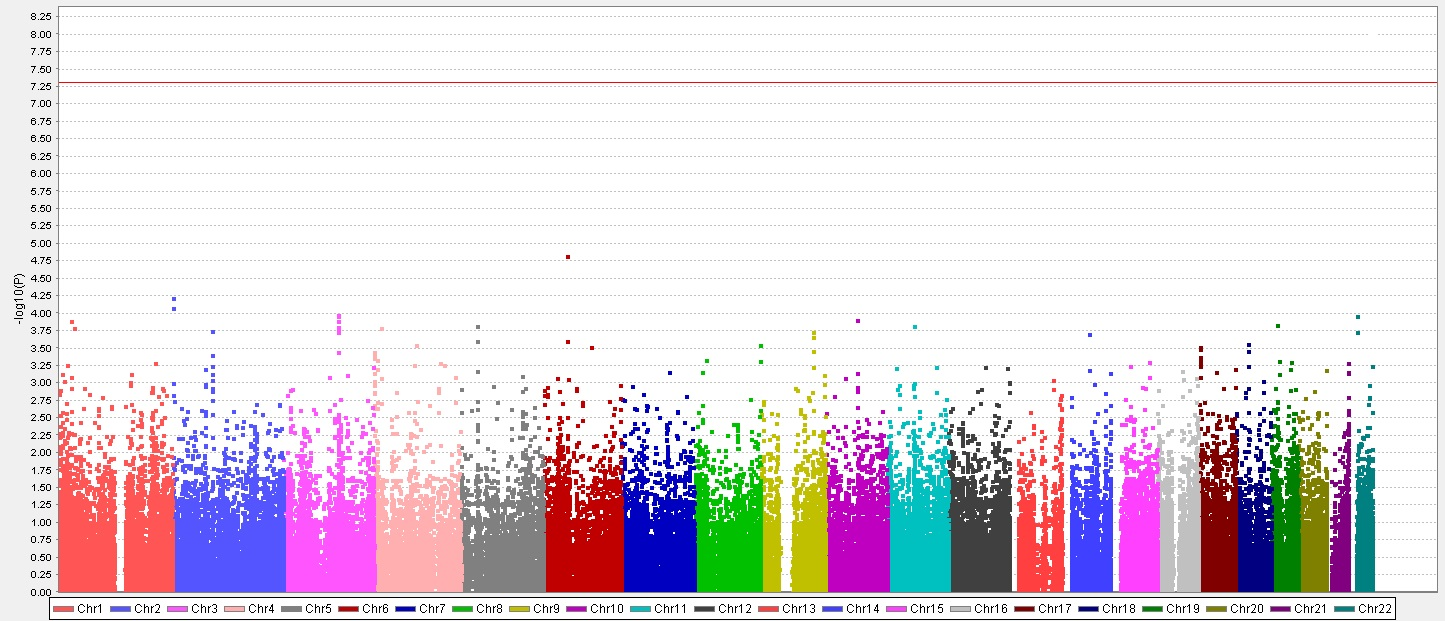

Supplement: S1 Fig — The–log10 p-values for each test are plotted against chromosomal position. A genome-wide significance threshold of 5 x 10^−8^ is indicated by the red horizontal bar. (TIF) [file pgen.1007667.s001.tif]

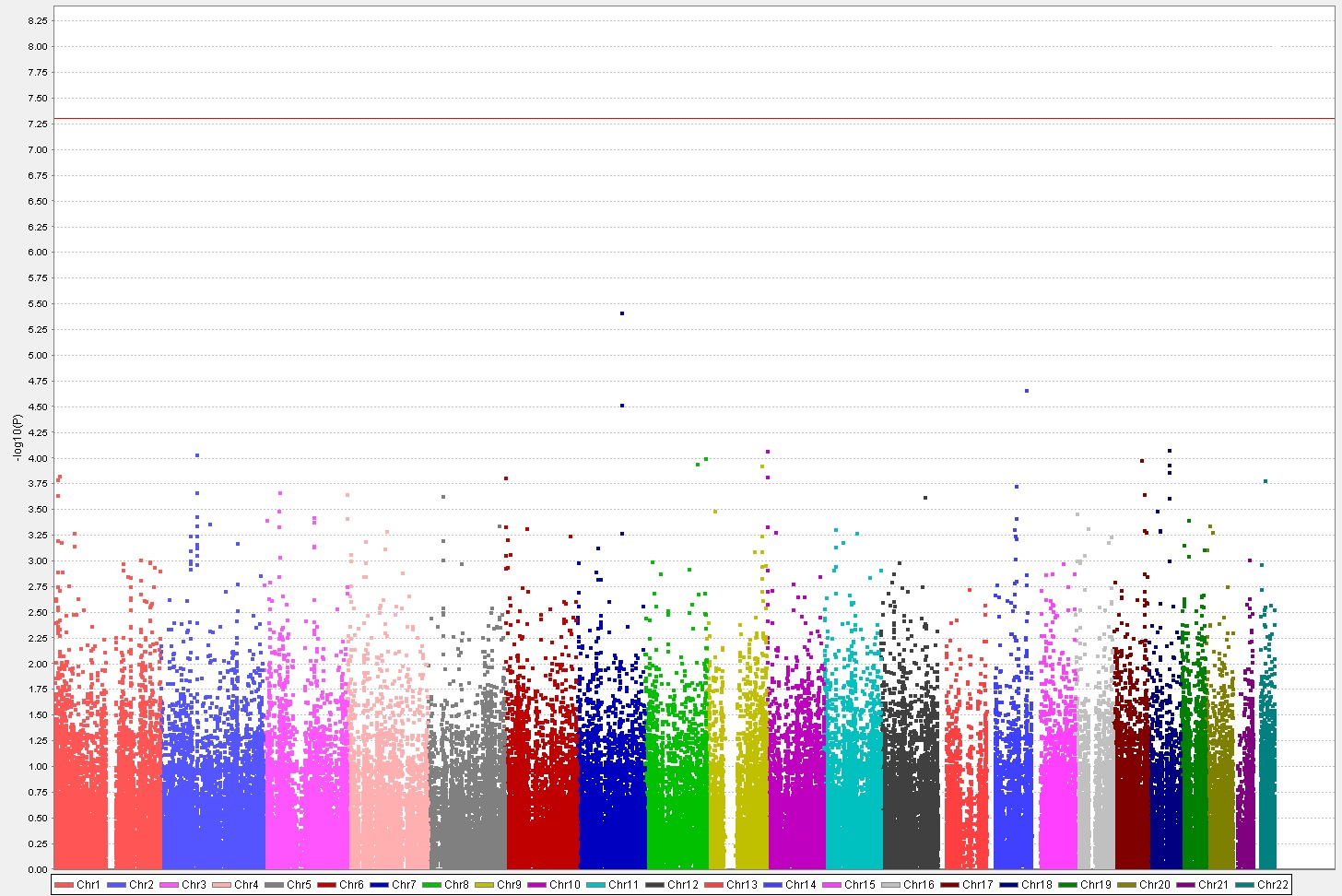

Supplement: S2 Fig — The–log10 p-values for each test are plotted against chromosomal position. A genome-wide significance threshold of 5 x 10^−8^ is indicated by the red horizontal bar. (TIF) [file pgen.1007667.s002.tif]

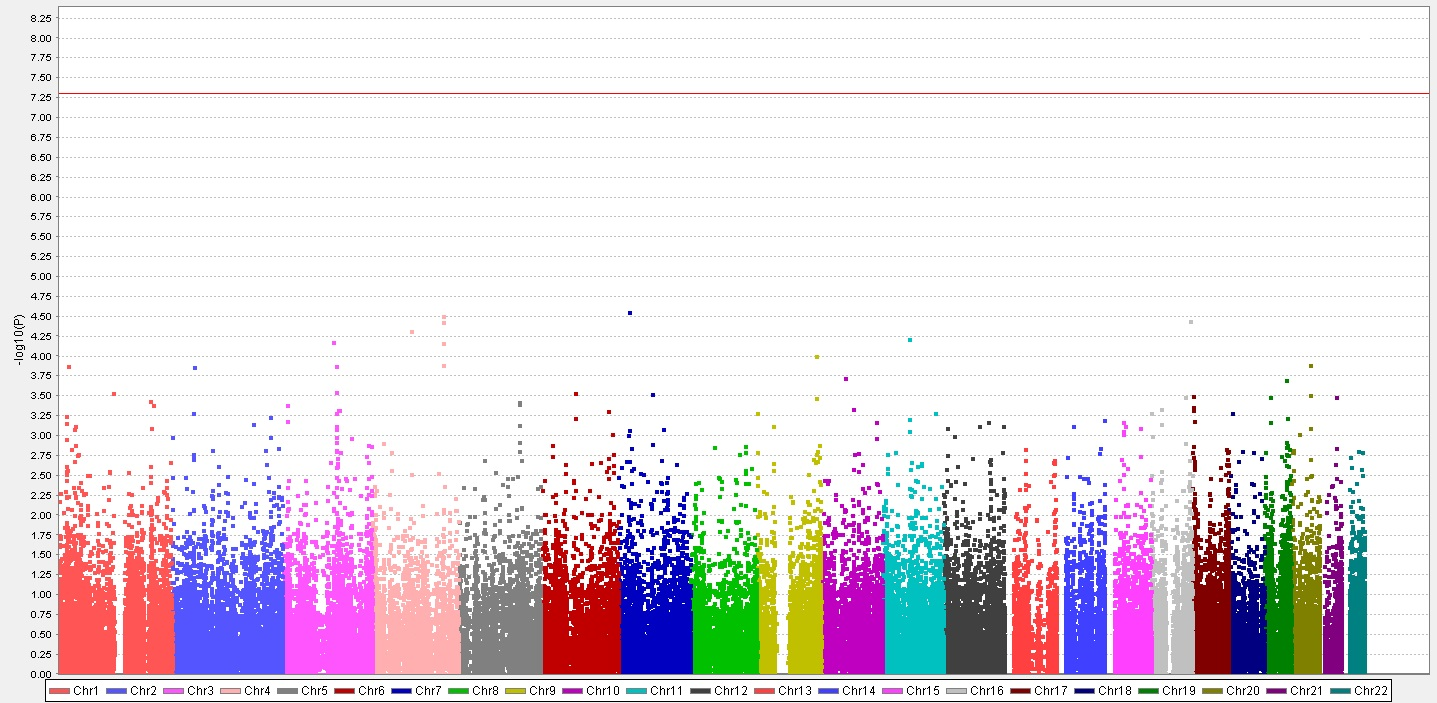

Supplement: S3 Fig — The -log10 p-values for each test are plotted against chromosomal position. A genome-wide significance threshold of 5 x 10^−8^ is indicated by the red horizontal bar. (TIF) [file pgen.1007667.s003.tif]

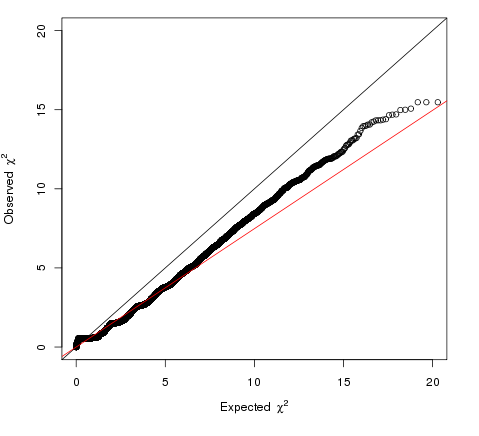

Supplement: S4 Fig — The black diagonal line indicates the expected distribution of test statistics under the null distribution. The red line indicates the linear trend of the ratio between observed and expected statistics. The plot indicates that overall test statistics are weaker than expected under the null hypothesis. (TIF) [file pgen.1007667.s004.tif]

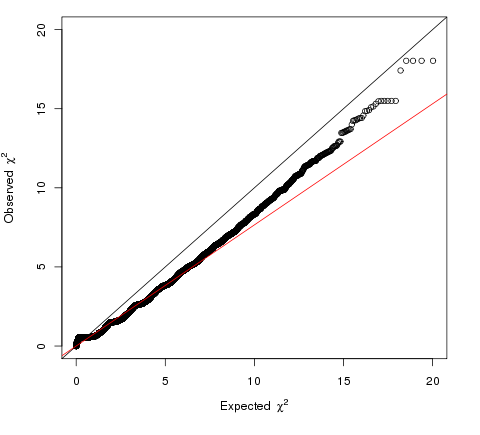

Supplement: S5 Fig — The black diagonal line indicates the expected distribution of test statistics under the null distribution. The red line indicates the linear trend of the ratio between observed and expected statistics. The plot indicates that overall test statistics are weaker than expected under the null hypothesis. (TIF) [file pgen.1007667.s005.tif]

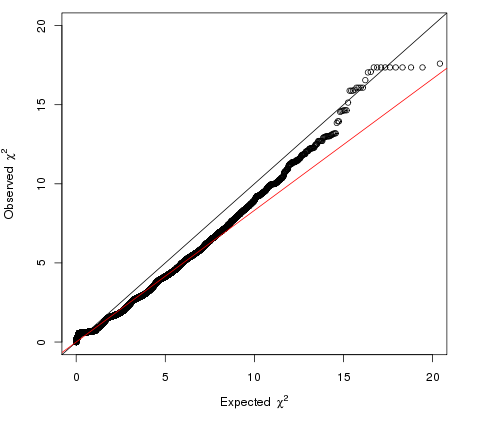

Supplement: S6 Fig — The black diagonal line indicates the expected distribution of test statistics under the null distribution. The red line indicates the linear trend of the ratio between observed and expected statistics. The plot indicates that overall test statistics are weaker than expected under the null hypothesis. (TIF) [file pgen.1007667.s006.tif]

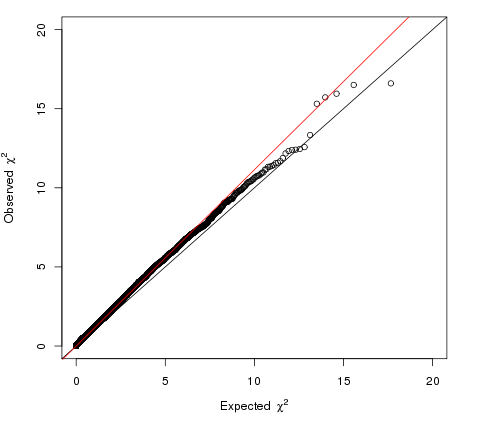

Supplement: S7 Fig — The black diagonal line indicates the expected distribution of test statistics under the null distribution. The red line indicates the linear trend of the ratio between observed and expected statistics. The plot shows little inflation of test statistics overall. (TIF) [file pgen.1007667.s007.tif]

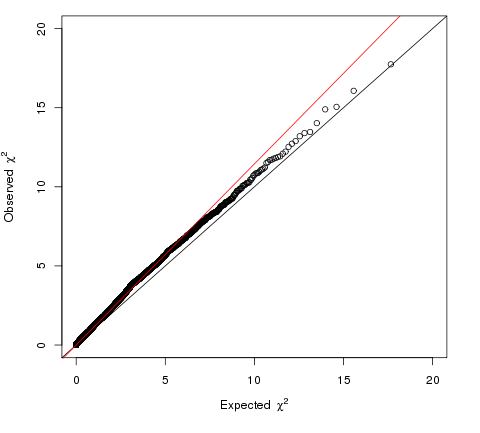

Supplement: S8 Fig — The black diagonal line indicates the expected distribution of test statistics under the null distribution. The red line indicates the linear trend of the ratio between observed and expected statistics. The plot shows little inflation of test statistics overall. (TIF) [file pgen.1007667.s008.tif]

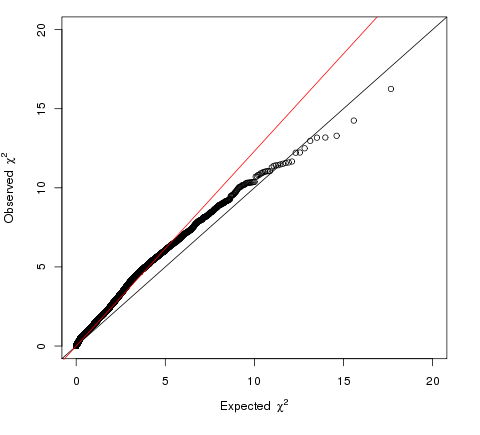

Supplement: S9 Fig — The black diagonal line indicates the expected distribution of test statistics under the null distribution. The red line indicates the linear trend of the ratio between observed and expected statistics. The plot shows slight inflation of test statistics at nominal significance levels, but less inflation of test statistics at higher significance levels. (TIF) [file pgen.1007667.s009.tif]

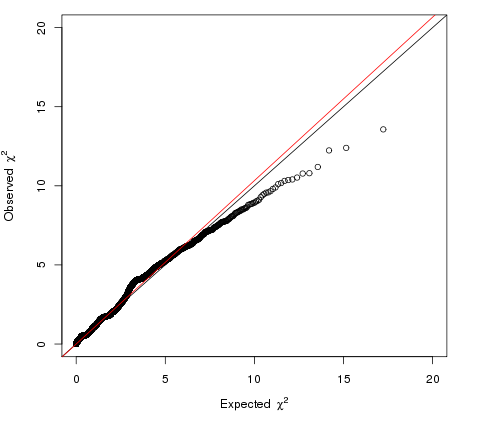

Supplement: S10 Fig — The black diagonal line indicates the expected distribution of test statistics under the null distribution. The red line indicates the linear trend of the ratio between observed and expected statistics. The plot shows little inflation of test statistics overall. (TIF) [file pgen.1007667.s010.tif]

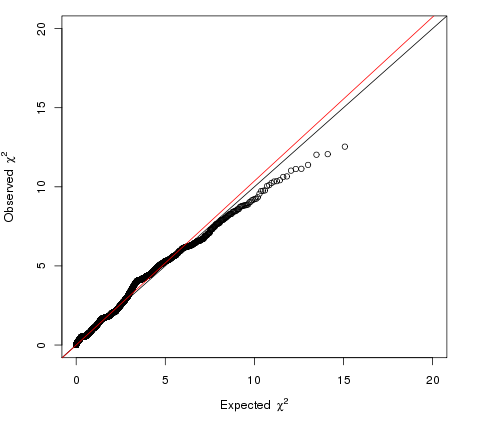

Supplement: S11 Fig — The black diagonal line indicates the expected distribution of test statistics under the null distribution. The red line indicates the linear trend of the ratio between observed and expected statistics. The plot shows little inflation of test statistics overall. (TIF) [file pgen.1007667.s011.tif]

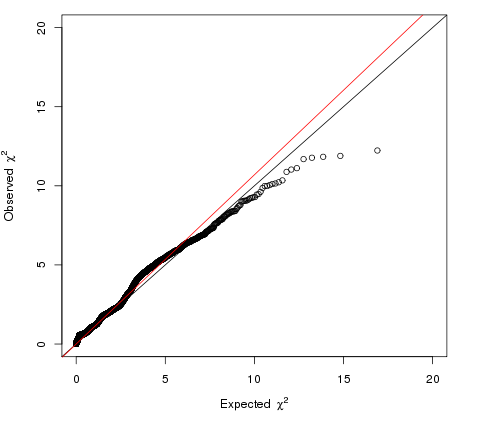

Supplement: S12 Fig — The black diagonal line indicates the expected distribution of test statistics under the null distribution. The red line indicates the linear trend of the ratio between observed and expected statistics. The plot shows little inflation of test statistics overall. (TIF) [file pgen.1007667.s012.tif]

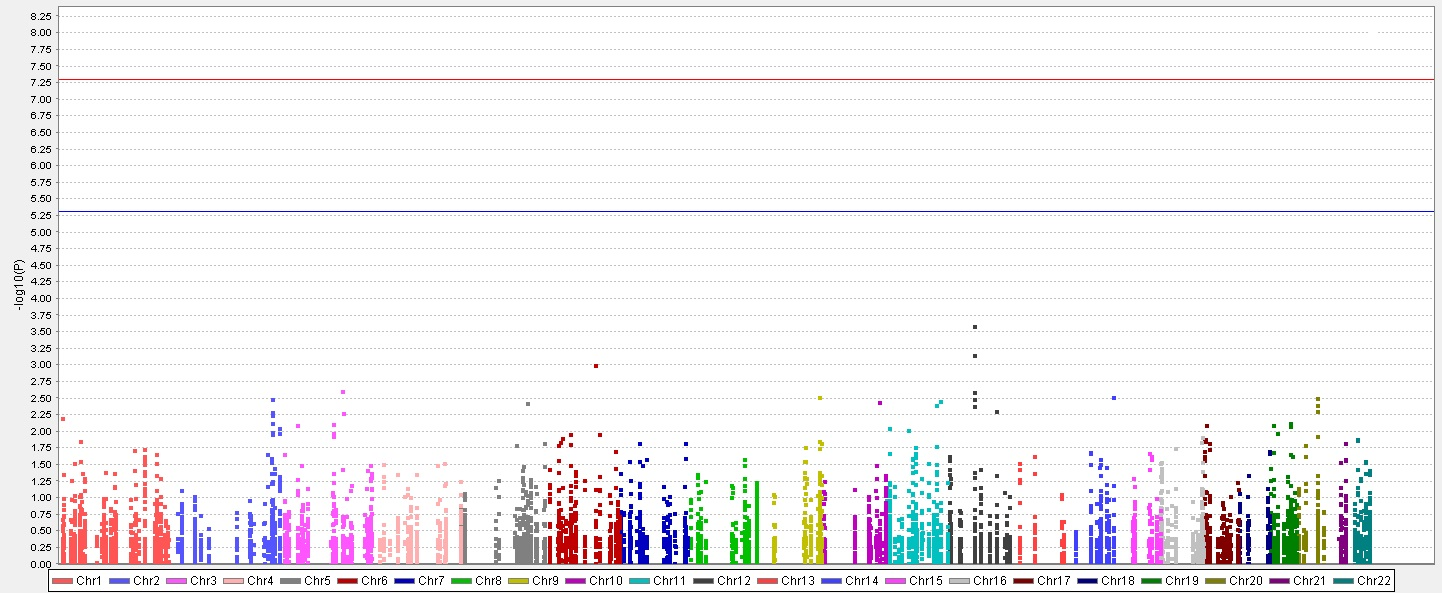

Supplement: S13 Fig — The–log10 p-values for each test are plotted against chromosomal position. A Bonferroni-corrected significance threshold of 4.5 x 10^−6^ is indicated by the blue horizontal bar; the traditional genome-wide significance threshold of 5 x 10^−8^ is indicated by the red horizontal bar. (TIF) [file pgen.1007667.s013.tif]

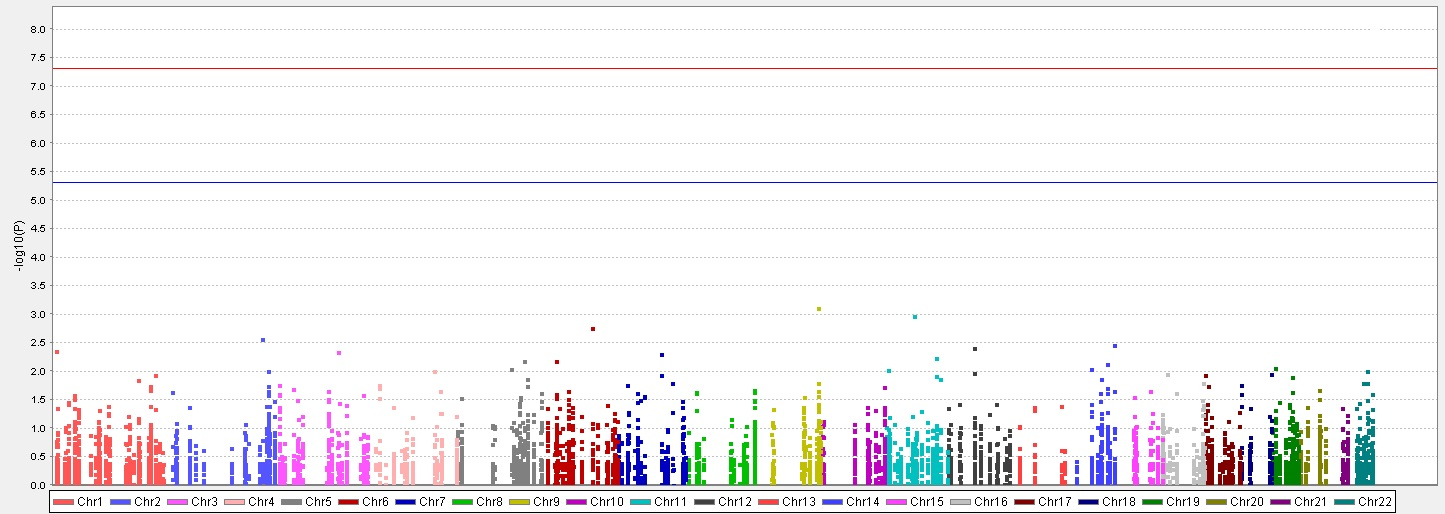

Supplement: S14 Fig — The–log10 p-values for each test are plotted against chromosomal position. A Bonferroni-corrected significance threshold of 4.5 x 10^−6^ is indicated by the blue horizontal bar; the traditional genome-wide significance threshold of 5 x 10^−8^ is indicated by the red horizontal bar. (TIF) [file pgen.1007667.s014.tif]

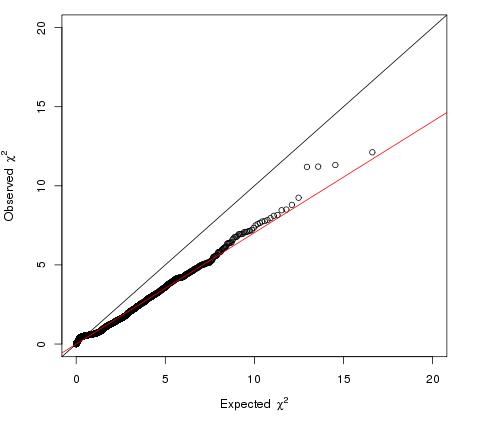

Supplement: S15 Fig — The black diagonal line indicates the expected distribution of test statistics under the null distribution. The red line indicates the linear trend of the ratio between observed and expected statistics. The plot indicates that overall test statistics are weaker than expected under the null hypothesis. (TIF) [file pgen.1007667.s015.tif]

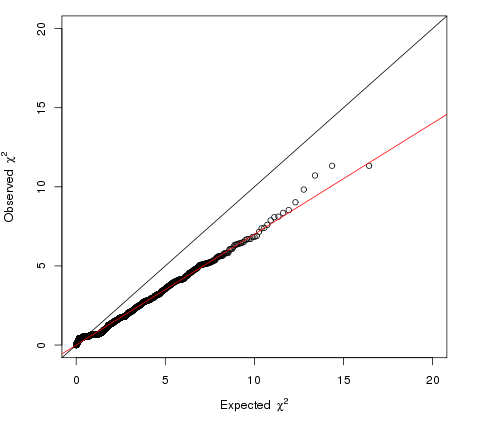

Supplement: S16 Fig — The black diagonal line indicates the expected distribution of test statistics under the null distribution. The red line indicates the linear trend of the ratio between observed and expected statistics. The plot indicates that overall test statistics are weaker than expected under the null hypothesis. (TIF) [file pgen.1007667.s016.tif]

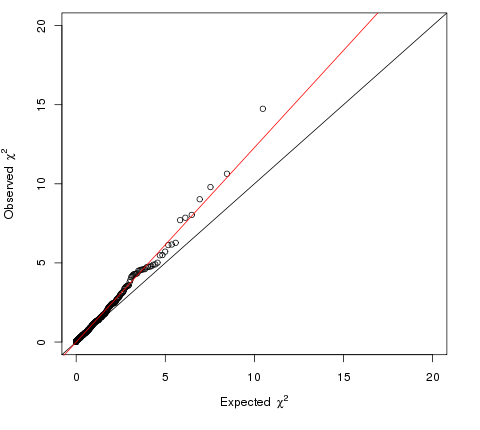

Supplement: S17 Fig — The black diagonal line indicates the expected distribution of test statistics under the null distribution. The red line indicates the linear trend of the ratio between observed and expected statistics. The plot shows a slight but acceptable inflation of nominally significant test statistics. (TIF) [file pgen.1007667.s017.tif]

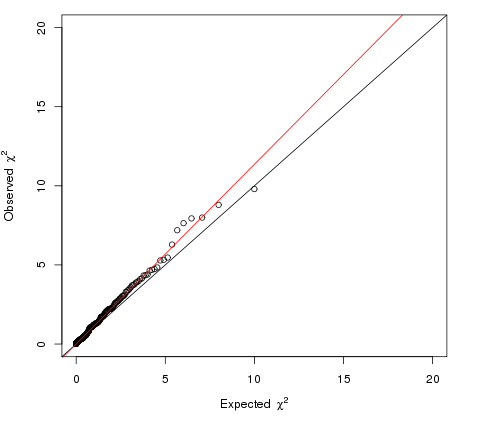

Supplement: S18 Fig — The black diagonal line indicates the expected distribution of test statistics under the null distribution. The red line indicates the linear trend of the ratio between observed and expected statistics. The plot shows little inflation of test statistics. (TIF) [file pgen.1007667.s018.tif]

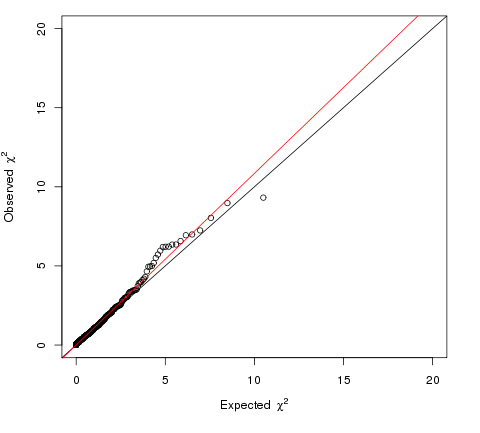

Supplement: S19 Fig — The black diagonal line indicates the expected distribution of test statistics under the null distribution. The red line indicates the linear trend of the ratio between observed and expected statistics. The plot shows little inflation of test statistics. (TIF) [file pgen.1007667.s019.tif]

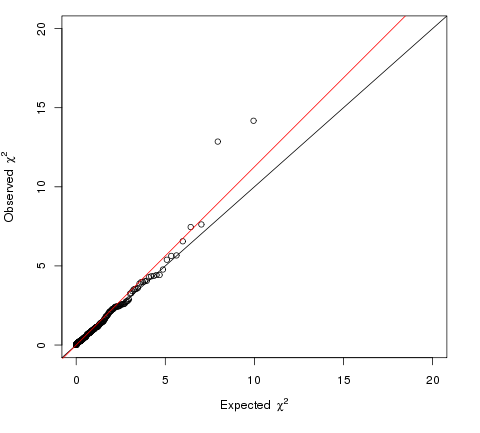

Supplement: S20 Fig — The black diagonal line indicates the expected distribution of test statistics under the null distribution. The red line indicates the linear trend of the ratio between observed and expected statistics. The plot shows little inflation of test statistics. (TIF) [file pgen.1007667.s020.tif]

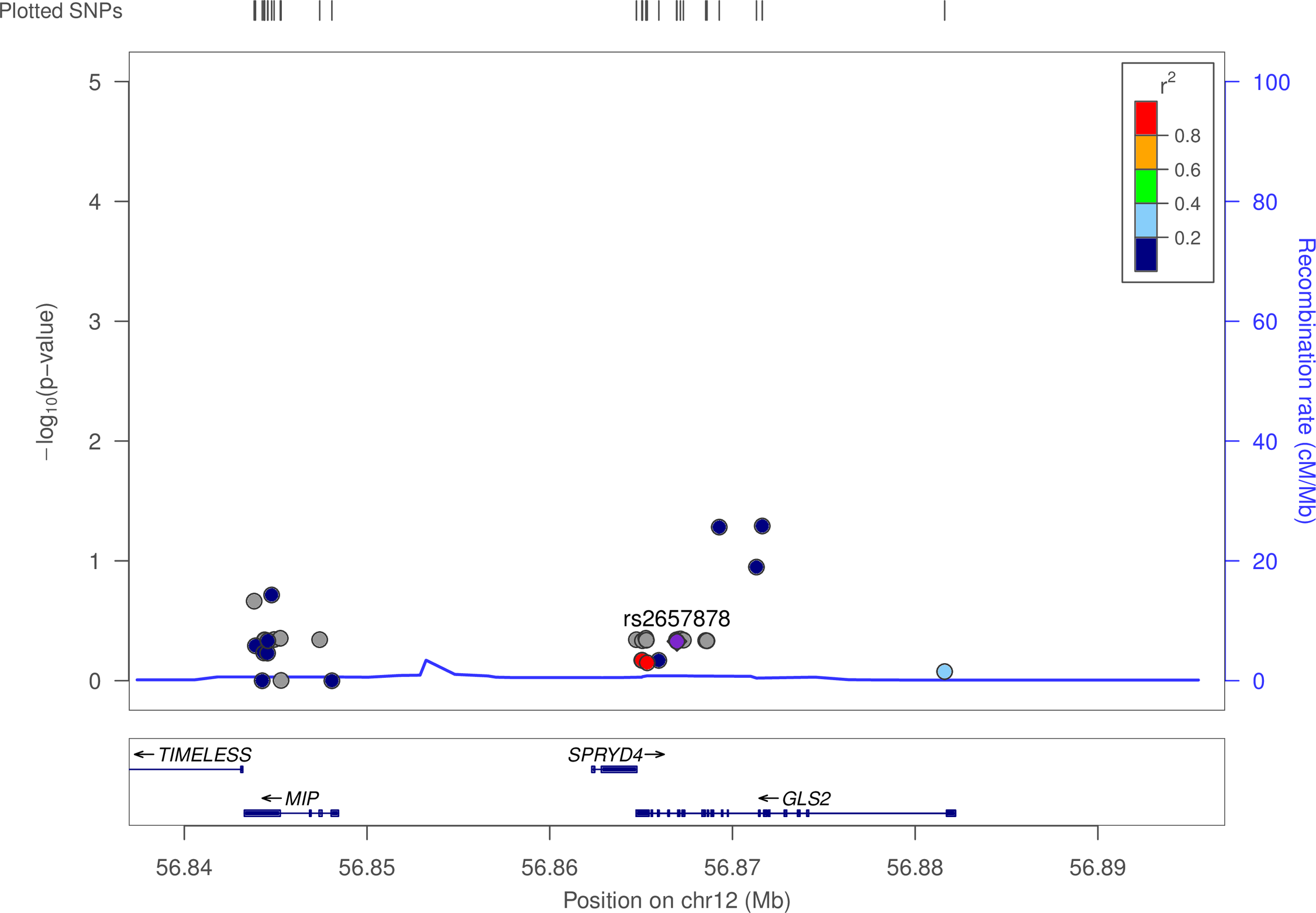

Supplement: S21 Fig — The–log10 p-values for individual SNV association tests are plotted against chromosomal position. Linkage disequilibrium is estimated from the 1000 Genomes 2014 European (EUR) sample. The strongest replication result at intronic variant rs2657878 is indicated by the purple diamond. No individual SNV tests are significant in GLS2 or surrounding genes. (TIF) [file pgen.1007667.s021.tif]
