# Supplementary material for: Human genetic variation in GLS2 is associated with development of complicated Staphylococcus aureus bacteremia
Source: PLoS Genet. 2018 Oct 5;14(10):e1007667. doi: 10.1371/journal.pgen.1007667 (PMC6192642; doi:10.1371/journal.pgen.1007667)
Supplement: S7 Table — Target regions spanned 2.05 Mb. Probes were selected from the Agilent SureSelect AllExon 50Mb + UTR v6 catalog, using the same parameters used to select that catalog and extending 10 bases from the ends of each. Targeted intervals are mapped to hg19 (GRCh37). (DOCX) [file pgen.1007667.s028.docx]

**S7 Table. Targeted capture array probe details for 342 target genes with SKAT-O association p<0.0035 in discovery sample analysis.**

| **Gene** | **Genomic interval (hg19)** | **Number of regions within target** | **Total size in base**  **pairs** | **% of targeted bases covered** | **Regions with coverage >=90%** | **Regions with coverage <90%** |
| --- | --- | --- | --- | --- | --- | --- |
| **ABCA4** | chr1:94458383-94586715 | 56 | 9806 | 95.1 | 51 | 5 |
| **ABCB11** | chr2:169778554-169887843 | 29 | 6213 | 90.4 | 27 | 2 |
| **ABCB6** | chr2:220074478-220083722 | 13 | 5485 | 89.1 | 12 | 1 |
| **ABHD13** | chr13:108870717-108886613 | 2 | 5378 | 100.0 | 2 | 0 |
| **ACKR4** | chr3:132316071-132337821 | 4 | 3031 | 60.3 | 3 | 1 |
| **ADM2** | chr22:50919975-50924879 | 2 | 4316 | 93.2 | 2 | 0 |
| **AGAP2** | chr12:58118066-58135954 | 20 | 6600 | 87.4 | 19 | 1 |
| **AGBL2** | chr11:47681133-47736951 | 20 | 5883 | 89.8 | 17 | 3 |
| **AHSG** | chr3:186330702-186339117 | 7 | 2493 | 87.1 | 6 | 1 |
| **AKR1C3** | chr10:5077536-5149888 | 14 | 4837 | 68.5 | 8 | 6 |
| **AKR1E2** | chr10:4828810-4890264 | 13 | 2678 | 85.8 | 11 | 2 |
| **ALS2CL** | chr3:46710475-46735204 | 21 | 9479 | 81.8 | 18 | 3 |
| **AMOTL2** | chr3:134074177-134094331 | 16 | 6834 | 76.8 | 9 | 7 |
| **ANKS6** | chr9:101493601-101559257 | 17 | 9146 | 88.8 | 14 | 3 |
| **ANKZF1** | chr2:220094469-220101401 | 6 | 6239 | 82.1 | 5 | 1 |
| **APOA4** | chr11:116691408-116694032 | 3 | 1531 | 100.0 | 3 | 0 |
| **ARHGAP9** | chr12:57866028-57882607 | 18 | 5496 | 96.5 | 17 | 1 |
| **ARV1** | chr1:231114717-231136489 | 8 | 2285 | 80.1 | 5 | 3 |
| **ATP13A5** | chr3:192992569-193096642 | 32 | 6868 | 69.4 | 30 | 2 |
| **ATP4B** | chr13:114303109-114312523 | 7 | 1640 | 100.0 | 7 | 0 |
| **AXDND1** | chr1:179334845-179523880 | 30 | 6689 | 81.7 | 26 | 4 |
| **B2M** | chr15:45003665-45011085 | 5 | 3251 | 84.8 | 2 | 3 |
| **BAAT** | chr9:104122689-104147297 | 5 | 3640 | 65.0 | 2 | 3 |
| **BAG3** | chr10:121410872-121437341 | 6 | 2867 | 97.0 | 5 | 1 |
| **BBOX1** | chr11:27062262-27149366 | 10 | 2664 | 97.6 | 9 | 1 |
| **BEND5** | chr1:49193185-49242651 | 6 | 2258 | 96.6 | 6 | 0 |
| **BRD8** | chr5:137475445-137514685 | 31 | 7670 | 88.5 | 25 | 6 |
| **BROX** | chr1:222885885-222908548 | 17 | 5320 | 80.2 | 13 | 4 |
| **BSDC1** | chr1:32830224-32860342 | 11 | 6965 | 85.6 | 8 | 3 |
| **C11ORF44** | chr11:130542841-130587257 | 3 | 3607 | 99.4 | 3 | 0 |
| **C14ORF79** | chr14:105452102-105476829 | 7 | 5365 | 90.9 | 6 | 1 |
| **C15ORF56** | chr15:40542855-40545180 | 1 | 2326 | 100.0 | 1 | 0 |
| **C15ORF60** | chr15:73735489-73852365 | 6 | 1047 | 100.0 | 6 | 0 |
| **C16ORF3** | chr16:90095306-90096319 | 1 | 1014 | 93.2 | 1 | 0 |
| **C17ORF67** | chr17:54869264-54916144 | 9 | 2590 | 88.2 | 8 | 1 |
| **C22ORF24** | chr22:32329497-32341514 | 3 | 1814 | 92.0 | 2 | 1 |
| **C22ORF42** | chr22:32544983-32555319 | 8 | 2215 | 83.2 | 6 | 2 |
| **C2ORF80** | chr2:209030057-209054807 | 10 | 1635 | 99.4 | 10 | 0 |
| **C3ORF67** | chr3:58703082-59035820 | 26 | 9975 | 72.3 | 23 | 3 |
| **C5AR1** | chr19:47793270-47825337 | 8 | 3142 | 73.5 | 2 | 6 |
| **C7ORF57** | chr7:48075098-48100911 | 10 | 2487 | 97.9 | 9 | 1 |
| **C7ORF69** | chr7:47834879-47859455 | 4 | 888 | 100.0 | 4 | 0 |
| **CA6** | chr1:9005883-9035161 | 10 | 2895 | 73.9 | 9 | 1 |
| **CABYR** | chr18:21718910-21741577 | 12 | 3457 | 77.2 | 7 | 5 |
| **CALR3** | chr19:16589858-16739025 | 16 | 3704 | 98.8 | 15 | 1 |
| **CAMTA1** | chr1:6845374-7829776 | 30 | 10633 | 93.4 | 27 | 3 |
| **CCDC108** | chr2:219867558-219906283 | 33 | 11203 | 96.4 | 32 | 1 |
| **CCDC25** | chr8:27590823-27630180 | 9 | 4609 | 81.4 | 6 | 3 |
| **CCDC28B** | chr1:32665977-32671001 | 6 | 2941 | 78.4 | 5 | 1 |
| **CCDC88B** | chr11:64107680-64125016 | 24 | 8816 | 78.0 | 22 | 2 |
| **CCDC92** | chr12:124403197-124457542 | 8 | 6680 | 88.7 | 4 | 4 |
| **CCNI** | chr4:77968301-77997168 | 10 | 3941 | 79.4 | 6 | 4 |
| **CCNJL** | chr5:159678649-159772413 | 13 | 4991 | 86.4 | 10 | 3 |
| **CD177** | chr19:43857801-43867490 | 10 | 2976 | 71.9 | 9 | 1 |
| **CD1A** | chr1:158223917-158228069 | 6 | 2216 | 100.0 | 6 | 0 |
| **CDC25B** | chr20:3767409-3786778 | 17 | 4880 | 91.6 | 14 | 3 |
| **CDHR3** | chr7:105517232-105676887 | 30 | 10394 | 40.2 | 19 | 11 |
| **CDK3** | chr17:73996977-74002090 | 8 | 1963 | 93.0 | 6 | 2 |
| **CEACAM20** | chr19:45005719-45033821 | 13 | 2456 | 88.2 | 11 | 2 |
| **CENPM** | chr22:42334715-42343178 | 7 | 1943 | 95.1 | 6 | 1 |
| **CES3** | chr16:66995122-67009062 | 13 | 5204 | 81.4 | 12 | 1 |
| **CGN** | chr1:151482976-151511178 | 23 | 7203 | 85.0 | 17 | 6 |
| **CHORDC1** | chr11:89933587-89956542 | 9 | 11566 | 65.6 | 6 | 3 |
| **CHRNA2** | chr8:27317268-27337410 | 10 | 5009 | 87.2 | 6 | 4 |
| **CHRNB4** | chr15:78916451-79020106 | 19 | 5356 | 57.4 | 8 | 11 |
| **CLDN14** | chr21:37832909-37948877 | 8 | 3013 | 84.3 | 5 | 3 |
| **CLEC3A** | chr16:78056402-78100668 | 6 | 3127 | 73.1 | 4 | 2 |
| **CNTN2** | chr1:205012315-205047637 | 21 | 10494 | 95.7 | 18 | 3 |
| **COL14A1** | chr8:121072009-121384285 | 55 | 9668 | 88.8 | 49 | 6 |
| **COMMD2** | chr3:149456247-149470296 | 4 | 4981 | 87.2 | 2 | 2 |
| **COX6C** | chr8:100885418-100906300 | 8 | 3241 | 50.9 | 4 | 4 |
| **CRISPLD2** | chr16:84853527-84954384 | 20 | 8418 | 68.4 | 13 | 7 |
| **CSDE1** | chr1:115259524-115301307 | 25 | 6278 | 81.8 | 19 | 6 |
| **CTSF** | chr11:66330924-66336322 | 11 | 3116 | 92.5 | 8 | 3 |
| **DCAF5** | chr14:69517588-69619924 | 12 | 8582 | 98.9 | 11 | 1 |
| **DDR1** | chr6:30844188-30867943 | 25 | 9627 | 84.4 | 19 | 6 |
| **DEFB1** | chr8:6728087-6735554 | 2 | 518 | 100.0 | 2 | 0 |
| **DES** | chr2:220283089-220291471 | 9 | 2644 | 100.0 | 9 | 0 |
| **DGKI** | chr7:137065773-137531848 | 41 | 17123 | 40.4 | 34 | 7 |
| **DOK3** | chr5:176928896-176938285 | 9 | 5044 | 81.2 | 6 | 3 |
| **DPY19L4** | chr8:95731921-95806086 | 20 | 7548 | 93.6 | 19 | 1 |
| **DPYD** | chr1:97543289-98386625 | 26 | 7086 | 89.9 | 24 | 2 |
| **DSEL** | chr18:65173809-65184227 | 2 | 9571 | 97.5 | 2 | 0 |
| **DST** | chr6:56322775-56819436 | 114 | 43733 | 84.6 | 100 | 14 |
| **DUSP3** | chr17:41843479-41856378 | 6 | 5144 | 85.6 | 3 | 3 |
| **DUSP6** | chr12:89740999-89747058 | 4 | 4712 | 99.5 | 3 | 1 |
| **E2F2** | chr1:23832910-23857722 | 7 | 5599 | 96.2 | 6 | 1 |
| **EFCAB4B** | chr12:3715789-3873995 | 23 | 12926 | 34.3 | 19 | 4 |
| **EHHADH** | chr3:184908402-184999788 | 10 | 4900 | 93.5 | 7 | 3 |
| **EIF3I** | chr1:32687519-32697215 | 11 | 1945 | 94.6 | 10 | 1 |
| **ELAC2** | chr17:12894919-12921514 | 20 | 7543 | 70.2 | 13 | 7 |
| **ELMOD1** | chr11:107461807-107537515 | 18 | 6216 | 61.7 | 14 | 4 |
| **ELSPBP1** | chr19:48497898-48528420 | 7 | 1262 | 100.0 | 7 | 0 |
| **EMC3** | chr3:10004211-10052810 | 11 | 4490 | 71.4 | 7 | 4 |
| **EML6** | chr2:54950626-55199167 | 42 | 15262 | 64.5 | 39 | 3 |
| **EPN3** | chr17:48609894-48621121 | 12 | 4882 | 93.6 | 10 | 2 |
| **EPT1** | chr2:26531405-26618769 | 12 | 8792 | 98.7 | 12 | 0 |
| **EXOC3** | chr5:443263-472062 | 16 | 8502 | 60.7 | 6 | 10 |
| **EXPH5** | chr11:108376148-108464475 | 10 | 11043 | 87.6 | 7 | 3 |
| **EYS** | chr6:64429866-66417128 | 54 | 18216 | 88.8 | 45 | 9 |
| **FAM111B** | chr11:58874648-58894898 | 4 | 3942 | 65.2 | 1 | 3 |
| **FAM151B** | chr5:79783778-79838392 | 11 | 3329 | 63.6 | 6 | 5 |
| **FAM153B** | chr5:175487682-177210409 | 80 | 20892 | 17.6 | 31 | 49 |
| **FAM201A** | chr9:38620461-38624997 | 4 | 3927 | 88.4 | 3 | 1 |
| **FAM207A** | chr21:46359915-46396914 | 8 | 1562 | 75.3 | 6 | 2 |
| **FAM221A** | chr7:23719723-23742878 | 10 | 7674 | 30.8 | 7 | 3 |
| **FAM92A1** | chr8:94710779-94743765 | 16 | 7740 | 82.8 | 10 | 6 |
| **FAT1** | chr4:187508927-187647886 | 32 | 16808 | 94.7 | 29 | 3 |
| **FBXO10** | chr9:37510879-37588881 | 15 | 6359 | 81.2 | 11 | 4 |
| **FBXO47** | chr17:37092675-37123665 | 11 | 2522 | 100.0 | 11 | 0 |
| **FGA** | chr4:155504268-155511928 | 6 | 4076 | 100.0 | 6 | 0 |
| **FGB** | chr4:155484098-155493925 | 8 | 4545 | 62.6 | 7 | 1 |
| **FGF8** | chr10:103529877-103540136 | 7 | 1543 | 93.9 | 6 | 1 |
| **FGG** | chr4:155525276-155534129 | 6 | 3720 | 87.8 | 5 | 1 |
| **FN1** | chr2:216225153-216300905 | 41 | 18135 | 67.4 | 33 | 8 |
| **FNDC7** | chr1:109255269-109285377 | 13 | 3663 | 100.0 | 13 | 0 |
| **FRMD5** | chr15:44162949-44487502 | 18 | 6934 | 92.7 | 14 | 4 |
| **FUBP1** | chr1:78409730-78444899 | 24 | 6244 | 64.4 | 20 | 4 |
| **GABRA1** | chr5:161274187-161326985 | 14 | 5774 | 100.0 | 14 | 0 |
| **GJA3** | chr13:20712384-20735198 | 2 | 5251 | 100.0 | 2 | 0 |
| **GLI3** | chr7:42000538-42277479 | 19 | 9620 | 93.9 | 14 | 5 |
| **GLS2** | chr12:56864718-56882208 | 18 | 4430 | 89.4 | 14 | 4 |
| **GNPDA1** | chr5:141371304-141392630 | 7 | 4115 | 93.9 | 6 | 1 |
| **GP5** | chr3:194115540-194120005 | 2 | 3603 | 96.0 | 1 | 1 |
| **GPC2** | chr7:99767219-99775059 | 9 | 4064 | 87.4 | 8 | 1 |
| **GPRC6A** | chr6:117113238-117150230 | 6 | 3002 | 100.0 | 6 | 0 |
| **GRAMD3** | chr5:125695778-125832196 | 20 | 5538 | 86.4 | 17 | 3 |
| **GTF2H4** | chr6:30875951-30881893 | 12 | 2593 | 100.0 | 12 | 0 |
| **HCCAT5** | chr16:73126238-73127683 | 3 | 1139 | 56.4 | 2 | 1 |
| **HDAC10** | chr22:50683602-50689844 | 9 | 5132 | 91.1 | 8 | 1 |
| **HECA** | chr6:139456239-139501956 | 4 | 5701 | 97.6 | 4 | 0 |
| **HIVEP1** | chr6:12008985-12165242 | 14 | 11255 | 95.9 | 11 | 3 |
| **HMCN2** | chr9:133028148-133309520 | 87 | 17374 | 94.2 | 85 | 2 |
| **HTN1** | chr4:70916109-70924575 | 4 | 3900 | 15.2 | 1 | 3 |
| **ICK** | chr6:52866067-52926610 | 15 | 6559 | 95.9 | 14 | 1 |
| **IFT172** | chr2:27667228-27712688 | 48 | 9383 | 95.5 | 44 | 4 |
| **IGSF10** | chr3:151143162-151176507 | 9 | 11917 | 98.4 | 7 | 2 |
| **IGSF11** | chr3:118619394-118864925 | 16 | 5203 | 81.7 | 8 | 8 |
| **IGSF3** | chr1:117117010-117210387 | 13 | 7661 | 98.2 | 11 | 2 |
| **IL20RA** | chr6:137321098-137366327 | 10 | 4930 | 84.9 | 6 | 4 |
| **IQCC** | chr1:32671226-32674298 | 5 | 2393 | 96.7 | 5 | 0 |
| **ITFG3** | chr16:284535-319952 | 20 | 5559 | 88.6 | 15 | 5 |
| **ITGB8** | chr7:20370315-20455392 | 14 | 15441 | 35.2 | 10 | 4 |
| **JPH2** | chr20:42740325-42816228 | 7 | 6083 | 100.0 | 7 | 0 |
| **KCNH8** | chr3:19189936-19577148 | 18 | 6592 | 90.8 | 16 | 2 |
| **KIAA0513** | chr16:85061347-85127846 | 14 | 9119 | 95.7 | 13 | 1 |
| **KIF14** | chr1:200520615-200589872 | 30 | 7877 | 98.3 | 30 | 0 |
| **KIF19** | chr17:72322339-72351969 | 20 | 6040 | 83.8 | 17 | 3 |
| **KLHDC4** | chr16:87730081-87799613 | 24 | 10134 | 46.3 | 11 | 13 |
| **KRIT1** | chr7:91828273-91875490 | 18 | 6564 | 85.4 | 16 | 2 |
| **KRTAP19-8** | chr21:32410468-32410805 | 1 | 338 | 100.0 | 1 | 0 |
| **KYNU** | chr2:143635057-143799900 | 20 | 3470 | 85.2 | 17 | 3 |
| **LCMT2** | chr15:43619964-43622830 | 1 | 2867 | 100.0 | 1 | 0 |
| **LINC00661** | chr19:16126087-16138282 | 13 | 4400 | 17.0 | 0 | 13 |
| **LOC100126784** | chr11:19732470-19736228 | 1 | 3759 | 36.9 | 0 | 1 |
| **LOC100129175** | chr2:219866927-219880454 | 3 | 2053 | 24.0 | 1 | 2 |
| **LOC100130691** | chr2:178148226-178257429 | 9 | 5386 | 11.1 | 4 | 5 |
| **LOC100287042** | chr17:73267370-73269986 | 1 | 2617 | 69.7 | 0 | 1 |
| **LOC101054525** | chr11:100999798-101030011 | 5 | 1323 | 62.9 | 1 | 4 |
| **LOC401324** | chr7:35353456-35416096 | 3 | 3689 | 23.2 | 0 | 3 |
| **LRMP** | chr12:25173926-25261279 | 34 | 5840 | 66.8 | 20 | 14 |
| **MACC1** | chr7:20174268-20257037 | 9 | 10078 | 92.8 | 7 | 2 |
| **MGME1** | chr20:17949546-17971775 | 5 | 2436 | 100.0 | 5 | 0 |
| **MICALL2** | chr7:1468091-1499148 | 11 | 9063 | 69.1 | 5 | 6 |
| **MIOS** | chr7:7606493-7648570 | 14 | 7094 | 59.0 | 8 | 6 |
| **MIR1283-1** | chr19:54191725-54191831 | 1 | 107 | 100.0 | 1 | 0 |
| **MIR2053** | chr8:113655712-113655822 | 1 | 111 | 100.0 | 1 | 0 |
| **MIR4741** | chr18:20513302-20513411 | 1 | 110 | 100.0 | 1 | 0 |
| **MND1** | chr4:154265791-154336280 | 11 | 1733 | 85.9 | 9 | 2 |
| **MRAP2** | chr6:84743410-84800615 | 4 | 2293 | 99.1 | 4 | 0 |
| **MST1** | chr3:49721370-49726944 | 6 | 5204 | 76.2 | 5 | 1 |
| **MYO1F** | chr19:8585664-8642471 | 26 | 9070 | 76.9 | 15 | 11 |
| **MYRFL** | chr12:70219074-70352887 | 25 | 4013 | 100.0 | 25 | 0 |
| **NAA15** | chr4:140222599-140341197 | 25 | 10212 | 90.0 | 20 | 5 |
| **NAP1L4** | chr11:2965650-3013617 | 23 | 6377 | 63.1 | 14 | 9 |
| **NDUFA4L2** | chr12:57628676-57634508 | 6 | 2926 | 61.7 | 3 | 3 |
| **NEK3** | chr13:52706765-52734006 | 16 | 4616 | 92.4 | 14 | 2 |
| **NFYB** | chr12:104510845-104532077 | 10 | 5156 | 88.8 | 9 | 1 |
| **NGEF** | chr2:233743386-233877992 | 19 | 5067 | 88.1 | 16 | 3 |
| **NKX2-6** | chr8:23559954-23564121 | 2 | 946 | 100.0 | 2 | 0 |
| **NOMO1** | chr16:14927528-14990027 | 33 | 6479 | 68.1 | 25 | 8 |
| **NPY6R** | chr5:137136872-137146450 | 2 | 3028 | 36.0 | 0 | 2 |
| **NR2C2** | chr3:14989081-15095117 | 20 | 12227 | 38.4 | 13 | 7 |
| **NRDE2** | chr14:90742570-90798491 | 17 | 7117 | 91.5 | 14 | 3 |
| **OPRM1** | chr6:154331621-154568011 | 15 | 21330 | 32.3 | 10 | 5 |
| **OR10Z1** | chr1:158576219-158577180 | 1 | 962 | 100.0 | 1 | 0 |
| **OR13C3** | chr9:107298020-107299147 | 1 | 1128 | 100.0 | 1 | 0 |
| **OR13J1** | chr9:35869250-35870608 | 1 | 1359 | 100.0 | 1 | 0 |
| **OR1F1** | chr16:3254237-3255198 | 1 | 962 | 100.0 | 1 | 0 |
| **OR5AK2** | chr11:56756337-56757352 | 1 | 1016 | 100.0 | 1 | 0 |
| **OR6V1** | chr7:142749407-142750454 | 1 | 1048 | 100.0 | 1 | 0 |
| **OSER1** | chr20:42824571-42839556 | 7 | 2522 | 81.7 | 6 | 1 |
| **OSR1** | chr2:19551236-19558424 | 4 | 5393 | 41.7 | 2 | 2 |
| **OVCH2** | chr11:7710659-7727978 | 18 | 2537 | 98.3 | 17 | 1 |
| **PAFAH2** | chr1:26286248-26324658 | 12 | 4075 | 92.4 | 11 | 1 |
| **PAK1** | chr11:77032742-77185690 | 24 | 6780 | 76.5 | 15 | 9 |
| **PAX3** | chr2:223064596-223163725 | 9 | 4814 | 99.3 | 8 | 1 |
| **PCSK4** | chr19:1481417-1490761 | 14 | 3987 | 95.1 | 12 | 2 |
| **PDGFRA** | chr4:55095254-55164424 | 29 | 10312 | 88.0 | 20 | 9 |
| **PDLIM5** | chr4:95372998-95589388 | 27 | 11775 | 86.5 | 20 | 7 |
| **PDZD9** | chr16:21995176-22012443 | 5 | 1433 | 100.0 | 5 | 0 |
| **PGGT1B** | chr5:114546517-114598579 | 10 | 3446 | 56.8 | 7 | 3 |
| **PGPEP1L** | chr15:99511449-99551034 | 6 | 1696 | 73.1 | 5 | 1 |
| **PGR** | chr11:100900345-101001265 | 8 | 15465 | 91.2 | 6 | 2 |
| **PHLPP1** | chr18:60382662-60647686 | 22 | 9603 | 73.7 | 16 | 6 |
| **PIGQ** | chr16:616985-634146 | 15 | 8922 | 83.4 | 10 | 5 |
| **PITPNM3** | chr17:6354573-6459887 | 22 | 11113 | 67.9 | 19 | 3 |
| **PLEC** | chr8:144989305-145050923 | 44 | 17882 | 100.0 | 44 | 0 |
| **PMVK** | chr1:154897198-154909494 | 5 | 1399 | 100.0 | 5 | 0 |
| **PNMAL2** | chr19:46990153-46999765 | 2 | 5546 | 96.1 | 1 | 1 |
| **POC5** | chr5:74969939-75013323 | 15 | 3755 | 81.0 | 11 | 4 |
| **POP4** | chr19:30094914-30108172 | 9 | 6772 | 78.4 | 3 | 6 |
| **POU5F1B** | chr8:128426525-128432321 | 2 | 5397 | 91.5 | 2 | 0 |
| **PPEF2** | chr4:76780991-76823734 | 19 | 5375 | 77.0 | 15 | 4 |
| **PPP1R15B** | chr1:204372482-204380955 | 2 | 5316 | 98.0 | 2 | 0 |
| **PPP1R26** | chr9:138370915-138380749 | 7 | 5416 | 94.7 | 6 | 1 |
| **PPP2R3A** | chr3:135684505-135866762 | 17 | 8198 | 93.7 | 16 | 1 |
| **PPP3CC** | chr8:22298322-22398667 | 16 | 3221 | 100.0 | 16 | 0 |
| **PPP5D1** | chr19:46984035-47104467 | 6 | 5831 | 39.3 | 1 | 5 |
| **PRM2** | chr16:11369483-11370347 | 2 | 814 | 100.0 | 2 | 0 |
| **PROSC** | chr8:37620091-37637296 | 7 | 3587 | 88.8 | 5 | 2 |
| **PSME3** | chr17:40976392-40995787 | 14 | 5445 | 89.7 | 11 | 3 |
| **PSMG3** | chr7:1606956-1610651 | 3 | 2035 | 89.9 | 2 | 1 |
| **PTPN14** | chr1:214522029-214725802 | 22 | 19275 | 24.8 | 17 | 5 |
| **PTPRF** | chr1:43990848-44089353 | 38 | 11499 | 85.4 | 31 | 7 |
| **PTTG2** | chr4:37962046-37962756 | 1 | 711 | 100.0 | 1 | 0 |
| **PYROXD2** | chr10:100143312-100174988 | 20 | 4304 | 66.5 | 15 | 5 |
| **QARS** | chr3:49133355-49142572 | 18 | 4945 | 96.0 | 17 | 1 |
| **RAD51** | chr15:40986962-41024366 | 13 | 3125 | 79.7 | 10 | 3 |
| **RAD54L** | chr1:46713350-46744155 | 18 | 3572 | 99.0 | 18 | 0 |
| **RASGEF1B** | chr4:82347537-82965407 | 19 | 10145 | 90.0 | 14 | 5 |
| **RBM38** | chr20:55966444-55984399 | 6 | 2815 | 100.0 | 6 | 0 |
| **RBMXL1** | chr1:89445129-89458653 | 3 | 5147 | 77.4 | 2 | 1 |
| **RIPPLY2** | chr6:84562975-84567244 | 4 | 896 | 100.0 | 4 | 0 |
| **ROCK1** | chr18:18526857-18691822 | 33 | 11724 | 56.2 | 27 | 6 |
| **RPL29** | chr3:52027606-52029968 | 3 | 1972 | 80.1 | 2 | 1 |
| **RPS10** | chr6:34385221-34393912 | 7 | 1793 | 56.6 | 5 | 2 |
| **RPS10-NUDT3** | chr6:34254963-34393912 | 9 | 2707 | 60.6 | 5 | 1 |
| **RSPH3** | chr6:159393893-159421229 | 8 | 6721 | 49.7 | 7 | 1 |
| **RUNX1** | chr21:36160088-37376975 | 28 | 16326 | 56.5 | 10 | 18 |
| **SAYSD1** | chr6:39071829-39082975 | 3 | 6488 | 37.9 | 2 | 1 |
| **SBF1** | chr22:50883419-50913510 | 38 | 10248 | 81.6 | 34 | 4 |
| **SCAMP2** | chr15:75136061-75165729 | 8 | 5173 | 70.9 | 5 | 3 |
| **SCNN1A** | chr12:6455999-6486906 | 17 | 6216 | 86.6 | 13 | 4 |
| **SDR39U1** | chr14:24908962-24912121 | 2 | 3148 | 91.7 | 1 | 1 |
| **SEC63** | chr6:108188950-108279492 | 23 | 9136 | 87.2 | 19 | 4 |
| **SEL1L2** | chr20:13829883-13977099 | 25 | 4387 | 73.6 | 20 | 5 |
| **SEMA5A** | chr5:9035128-9546243 | 26 | 12663 | 99.0 | 25 | 1 |
| **SEPT8** | chr5:132086499-132142943 | 17 | 8048 | 89.0 | 13 | 4 |
| **SERPINA5** | chr14:95027769-95059467 | 9 | 3955 | 82.1 | 7 | 2 |
| **SERPINE1** | chr7:100770360-100782557 | 9 | 3370 | 100.0 | 9 | 0 |
| **SERTAD1** | chr19:40927489-40931942 | 2 | 2154 | 87.5 | 1 | 1 |
| **SGOL2** | chr2:201374721-201448828 | 13 | 6729 | 76.8 | 6 | 7 |
| **SH3TC2** | chr5:148303192-148442747 | 20 | 34656 | 24.5 | 12 | 8 |
| **SIDT1** | chr3:113251133-113348435 | 28 | 8632 | 85.4 | 20 | 8 |
| **SIPA1L3** | chr19:38397851-38699022 | 28 | 10904 | 82.7 | 19 | 9 |
| **SIRT4** | chr12:120740109-120751062 | 4 | 1302 | 100.0 | 4 | 0 |
| **SLC1A7** | chr1:53552841-53608314 | 11 | 4496 | 93.4 | 10 | 1 |
| **SLC25A12** | chr2:172639905-172864776 | 24 | 6678 | 66.1 | 19 | 5 |
| **SLC25A16** | chr10:70237746-70287290 | 11 | 8084 | 26.2 | 8 | 3 |
| **SLC2A6** | chr9:136336206-136344286 | 8 | 3718 | 91.8 | 6 | 2 |
| **SLC3A2** | chr11:62623474-62656365 | 15 | 5576 | 74.7 | 8 | 7 |
| **SLC6A9** | chr1:44457162-44497174 | 18 | 5544 | 89.6 | 16 | 2 |
| **SLC9C1** | chr3:111859724-112013115 | 29 | 4830 | 100.0 | 29 | 0 |
| **SLIT1** | chr10:98757785-98945693 | 45 | 10180 | 92.9 | 39 | 6 |
| **SMARCA5** | chr4:144434606-144478652 | 24 | 8868 | 92.8 | 23 | 1 |
| **SMCHD1** | chr18:2655727-2805025 | 51 | 13230 | 81.7 | 43 | 8 |
| **SMYD4** | chr17:1682769-1733938 | 14 | 7057 | 67.5 | 7 | 7 |
| **SOD3** | chr4:24791524-24802477 | 4 | 2211 | 71.0 | 2 | 2 |
| **SPINK5** | chr5:147405236-147516935 | 39 | 5234 | 94.2 | 36 | 3 |
| **SPNS3** | chr17:4336973-4391513 | 13 | 2944 | 85.9 | 11 | 2 |
| **SPRR2F** | chr1:153084580-153086001 | 2 | 721 | 87.1 | 1 | 1 |
| **SPRY4** | chr5:141689982-141706030 | 5 | 6045 | 83.8 | 3 | 2 |
| **SPSB4** | chr3:140770234-140867463 | 7 | 3880 | 79.7 | 3 | 4 |
| **SRSF4** | chr1:29474240-29508647 | 10 | 3554 | 76.5 | 6 | 4 |
| **SSTR3** | chr22:37600267-37608426 | 3 | 4635 | 97.9 | 2 | 1 |
| **ST5** | chr11:8714888-8932508 | 46 | 15187 | 59.8 | 29 | 17 |
| **ST7-OT4** | chr7:116593943-116738870 | 8 | 2824 | 82.2 | 6 | 2 |
| **STX2** | chr12:131274135-131323829 | 12 | 3971 | 97.4 | 12 | 0 |
| **STX6** | chr1:180941840-180992267 | 9 | 6655 | 82.6 | 6 | 3 |
| **SUZ12** | chr17:30264027-30328074 | 17 | 8030 | 65.7 | 11 | 6 |
| **SYNGR3** | chr16:2039651-2044286 | 6 | 3540 | 96.9 | 5 | 1 |
| **TAGAP** | chr6:159455490-159466194 | 10 | 4398 | 100.0 | 10 | 0 |
| **TAPT1-AS1** | chr4:16228276-16321773 | 5 | 4292 | 24.6 | 1 | 4 |
| **TATDN2** | chr3:10289697-10327490 | 10 | 5825 | 100.0 | 10 | 0 |
| **TBX1** | chr22:19744216-19771126 | 12 | 3734 | 94.4 | 10 | 2 |
| **TCF20** | chr22:42556009-42739632 | 6 | 7668 | 97.9 | 5 | 1 |
| **TCF7** | chr5:133450392-133487566 | 12 | 10303 | 73.1 | 6 | 6 |
| **TCF7L2** | chr10:114709999-114927447 | 19 | 5429 | 94.0 | 16 | 3 |
| **TCP11L1** | chr11:33060953-33127499 | 14 | 5650 | 81.2 | 12 | 2 |
| **TET1** | chr10:70320107-70454249 | 12 | 9824 | 98.3 | 11 | 1 |
| **TEX36** | chr10:127265081-127371723 | 6 | 1428 | 100.0 | 6 | 0 |
| **TGFB1** | chr19:41807482-41859848 | 8 | 3420 | 97.3 | 7 | 1 |
| **TIAL1** | chr10:121332968-121356551 | 15 | 5991 | 65.5 | 7 | 8 |
| **TICRR** | chr15:90118703-90174297 | 23 | 8861 | 89.1 | 22 | 1 |
| **TIPRL** | chr1:168148073-168171361 | 8 | 3467 | 57.3 | 6 | 2 |
| **TM7SF3** | chr12:27124496-27167377 | 15 | 5810 | 59.3 | 11 | 4 |
| **TMEM260** | chr14:56955062-57117334 | 22 | 8517 | 69.8 | 16 | 6 |
| **TMEM39B** | chr1:32537622-32568477 | 15 | 3764 | 66.3 | 9 | 6 |
| **TMEM62** | chr15:43415467-43477354 | 17 | 5150 | 69.8 | 9 | 8 |
| **TMEM63B** | chr6:44094641-44123266 | 28 | 5525 | 86.3 | 24 | 4 |
| **TMEM63C** | chr14:77582901-77725848 | 28 | 6923 | 93.9 | 25 | 3 |
| **TMEM81** | chr1:205052247-205053655 | 1 | 1409 | 100.0 | 1 | 0 |
| **TMEM9B** | chr11:8968738-8986568 | 8 | 3317 | 94.0 | 7 | 1 |
| **TNFAIP8** | chr5:118604377-118735393 | 6 | 9715 | 47.1 | 4 | 2 |
| **TNKS1BP1** | chr11:57067093-57092436 | 14 | 7438 | 92.6 | 11 | 3 |
| **TP53BP2** | chr1:223967585-224033684 | 20 | 6397 | 89.5 | 17 | 3 |
| **TRABD2A** | chr2:85048764-85134142 | 14 | 9051 | 32.3 | 7 | 7 |
| **TRAT1** | chr3:108541535-108573862 | 6 | 2648 | 87.8 | 4 | 2 |
| **TRIM17** | chr1:228595626-228604593 | 6 | 3694 | 99.6 | 6 | 0 |
| **TRIQK** | chr8:93895748-94029911 | 12 | 6980 | 66.9 | 6 | 6 |
| **TRMT12** | chr8:125463038-125474401 | 2 | 2384 | 94.0 | 1 | 1 |
| **TRPM2** | chr21:45770036-45862974 | 35 | 7408 | 99.2 | 34 | 1 |
| **TTC16** | chr9:130478335-130493889 | 11 | 6590 | 64.0 | 10 | 1 |
| **TTC40** | chr10:134621886-134756337 | 59 | 11741 | 98.1 | 58 | 1 |
| **TUB** | chr11:8040781-8127669 | 15 | 7022 | 100.0 | 15 | 0 |
| **TUBD1** | chr17:57936831-57970316 | 12 | 3572 | 78.8 | 8 | 4 |
| **TXNRD1** | chr12:104609547-104744095 | 27 | 7250 | 89.5 | 21 | 6 |
| **TYRO3** | chr15:41849863-41871546 | 20 | 7109 | 79.6 | 17 | 3 |
| **UGCG** | chr9:114659036-114697664 | 10 | 4820 | 92.0 | 7 | 3 |
| **UPB1** | chr22:24863196-24924368 | 13 | 7319 | 58.2 | 8 | 5 |
| **USP18** | chr22:18632656-18660174 | 11 | 2349 | 78.6 | 10 | 1 |
| **VANGL2** | chr1:160370354-160398478 | 8 | 5512 | 100.0 | 8 | 0 |
| **WDR3** | chr1:118472333-118509175 | 28 | 10835 | 58.5 | 26 | 2 |
| **WDR92** | chr2:68350058-68384702 | 11 | 4303 | 80.2 | 7 | 4 |
| **WFDC10B** | chr20:44313280-44333668 | 4 | 712 | 100.0 | 4 | 0 |
| **XIRP1** | chr3:39224691-39234097 | 2 | 6507 | 100.0 | 2 | 0 |
| **YLPM1** | chr14:75230059-75322254 | 23 | 11210 | 80.9 | 18 | 5 |
| **ZC3H10** | chr12:56511933-56516290 | 2 | 3488 | 77.8 | 1 | 1 |
| **ZC3H12D** | chr6:149768756-149806207 | 8 | 6058 | 53.3 | 7 | 1 |
| **ZC3HAV1** | chr7:138728256-138794475 | 13 | 8495 | 98.7 | 13 | 0 |
| **ZNF224** | chr19:44598472-44612489 | 6 | 5674 | 56.4 | 3 | 3 |
| **ZNF229** | chr19:44921675-44952776 | 8 | 5668 | 92.6 | 6 | 2 |
| **ZNF317** | chr19:9251046-9274110 | 6 | 5185 | 91.6 | 5 | 1 |
| **ZNF367** | chr9:99148213-99180679 | 5 | 3804 | 100.0 | 5 | 0 |
| **ZNF460** | chr19:57791409-57805446 | 5 | 4104 | 86.9 | 3 | 2 |
| **ZNF479** | chr7:57187311-57207581 | 5 | 2172 | 85.5 | 2 | 3 |
| **ZNF48** | chr16:30389417-30411439 | 4 | 3824 | 100.0 | 4 | 0 |
| **ZNF639** | chr3:179040769-179053333 | 11 | 4031 | 84.5 | 6 | 5 |
| **ZNF646** | chr16:31085733-31095527 | 2 | 8675 | 99.2 | 1 | 1 |
| **ZNF766** | chr19:52772814-52795987 | 8 | 4175 | 85.4 | 5 | 3 |
| **ZNRD1-AS1** | chr6:29968778-30029427 | 13 | 19791 | 9.2 | 1 | 12 |
